# New Insights Into the Anatomy, Connectivity and Clinical Implications of the Middle Longitudinal Fasciculus

**DOI:** 10.3389/fnana.2020.610324

**Published:** 2021-01-29

**Authors:** Francesco Latini, Gianluca Trevisi, Markus Fahlström, Malin Jemstedt, Åsa Alberius Munkhammar, Maria Zetterling, Göran Hesselager, Mats Ryttlefors

**Affiliations:** ^1^Section of Neurosurgery, Department of Neuroscience, Uppsala University, Uppsala, Sweden; ^2^Neurosurgical Unit, Department of Surgery, Ospedale Santo Spirito, Pescara, Italy; ^3^Section of Radiology, Department of Surgical Sciences, Uppsala University, Uppsala, Sweden; ^4^Section of Speech-Language Pathology, Department of Neuroscience, Uppsala University, Uppsala, Sweden; ^5^Rehabilitation and Pain Centre, Uppsala University Hospital, Uppsala, Sweden

**Keywords:** diffusion tensor tractography, verbal memory, visual-auditory integration, Human Connectome Project, verbal learning, auditory hallucinations, white matter, MdLF

## Abstract

The middle longitudinal fascicle (MdLF) is a long, associative white matter tract connecting the superior temporal gyrus (STG) with the parietal and occipital lobe. Previous studies show different cortical terminations, and a possible segmentation pattern of the tract. In this study, we performed a post-mortem white matter dissection of 12 human hemispheres and an *in vivo* deterministic fiber tracking of 24 subjects acquired from the Human Connectome Project to establish whether a constant organization of fibers exists among the MdLF subcomponents and to acquire anatomical information on each subcomponent. Moreover, two clinical cases of brain tumors impinged on MdLF territories are reported to further discuss the anatomical results in light of previously published data on the functional involvement of this bundle. The main finding is that the MdLF is consistently organized into two layers: an antero-ventral segment (aMdLF) connecting the anterior STG (including temporal pole and planum polare) and the extrastriate lateral occipital cortex, and a posterior-dorsal segment (pMdLF) connecting the posterior STG, anterior transverse temporal gyrus and planum temporale with the superior parietal lobule and lateral occipital cortex. The anatomical connectivity pattern and quantitative differences between the MdLF subcomponents along with the clinical cases reported in this paper support the role of MdLF in high-order functions related to acoustic information. We suggest that pMdLF may contribute to the learning process associated with verbal-auditory stimuli, especially on left side, while aMdLF may play a role in processing/retrieving auditory information already consolidated within the temporal lobe.

## Introduction

In the last decade, a renewed interest in white matter tracts studied both *in vivo* [“virtual” dissection by diffusion tensor magnetic resonance imaging (MRI) -DTI- and intraoperative electrical stimulation] and *post-mortem* [different modifications of the Klinger technique (Agrawal et al., 2011) in most studies] has led to an improved understanding of the morphology and function of the complex system of human associative fibers involved in several higher neurocognitive functions.

The middle longitudinal fascicle (MdLF) is classically defined as an associative longitudinal fiber bundle connecting temporal, parietal, and occipital lobes. This white matter pathway has received less attention and is one of the most puzzling bundles because its morphology and function are still poorly understood.

Regarding morphology, the parietal cortical terminations have been the object of debate and some controversial results between post-mortem and *in vivo* studies are reported. This may be partially due to the fact that MdLF was first recognized in monkeys, where it interconnects the superior temporal gyrus (STG) with the inferior parietal lobule (IPL) (Seltzer and Pandya, 1984). However, fiber dissection studies in humans showed that MdLF parietal terminations are almost exclusively located in the superior parietal lobule (SPL) (Maldonado et al., 2013; Wang et al., 2013). Interestingly, both areas are cytoarchitectonically classified as Brodmann area 7 in the respective species and are functionally homologous although anatomically different (Wang et al., 2013). Nonetheless, DTI studies in humans have shown cortical terminations also at the level of the IPL, namely at both the angular (AG) and supramarginal (SMG) gyri (Menjot de Champfleur et al., 2013; Makris et al., 2017).

Moreover, in humans, posterior terminations of MdLF are not limited to the parietal lobe, as they are also directed toward the extrastriate occipital cortex (lateral occipital cortex, LOC) and cuneus (Cu). This occipital terminations of the MdLF have not been described in monkeys indeed (Bryant et al., 2020).

Middle longitudinal fascicle anterior terminations at the level of the STG and temporal pole (TP) are also controversial, as some authors found these terminations to reach the most anterior cortex of STG and TP (Makris et al., 2013a, b; Wang et al., 2013; Kalyvas et al., 2020), while others described a more posterior origin of MdLF fibers (Conner et al., 2018) or at least a less organized bundle with less numerous fibers at the level of TP (Maldonado et al., 2013).

Finally, there is a lack of agreement regarding MdLF segmentation. While DTI studies showed several subcomponents (up to six) of the MdLF (Makris et al., 2017), white matter dissection studies showed either a single bundle from the STG to posterior terminations or two components with a deeper layer of fibers originating more anteriorly and suggesting a segmentation pattern in the MdLF (Maldonado et al., 2013; Wang et al., 2013). More recently three subcomponents with different connection patterns have been described by Kalyvas et al. (2020).

The functional role of the MdLF is even less understood than its morphology. Some authors, on the basis of the cortical terminations, speculated that this bundle might be a connection between the dorsal and ventral stream of the language network (Saur et al., 2008), while others identified the MdLF as the potential pathway for the dorsal auditory stream (Wang et al., 2013; Kalyvas et al., 2020), as well as for ventral auditory stream. Others have speculated that functions are related to language comprehension, visuospatial and attention functions, and integration between auditory and visual information (Makris et al., 2017; Kalyvas et al., 2020).

However, cortical stimulation during awake surgery (De Witt Hamer et al., 2011) or lesion models, such as after post-operative resection of the bundle, have not provided solid evidence regarding its involvement in language, attention, or auditory functions. Nonetheless, recent data showed loss of symmetry of the DTI metrics of MdLF in patient affected by primary progressive aphasia compared to matched healthy controls, with a clear left-lateralized pattern of abnormality (Luo et al., 2019).

The first aim of this study was to investigate the MdLF anatomical connectivity between temporal, parietal and occipital regions using white matter fiber dissections of post-mortem human brains. Second, the analysis of the connectivity patterns of the MdLF was used to acquire anatomical information on MdLF subcomponent derived from *in vivo* DTI tracking. In addition, we aimed to discuss quantitative and anatomical results of this bundle in two clinical cases of tumors that involved the MdLF.

## Experimental Procedure

### Fiber Dissection Technique

#### Acquisition of Cadaver Specimens

Twelve normal cerebral hemispheres (six right and six left) obtained from six human cadavers (three females and three males; mean age 72 ± 6) donated to the Department of Medical Cell Biology, Section for Anatomy Studies at Uppsala University, Sweden, were studied. All individuals had no history of neurological diseases and death cause was not related to brain diseases. All donating individuals had given written consent for use of the whole cadaver for biomedical research and education in a testimonial donation letter. The study protocol was filed with the application for ethical vetting of research involving humans to the Regional Ethical Vetting Board in Uppsala, Sweden (Dnr 2014/468).

#### Perfusion Protocol and Specimen Preparation

Each brain was fixed with an intra-carotidal injection of 12% formalin within the first week after death. The brains were carefully extracted and put in 10% formalin for 24 h. The pia mater, arachnoid membrane and vascular structures were then carefully removed under microscopic magnification and the hemispheres were frozen at −15 to −20°C for 6–10 days, then slowly defrosted for 12 h (Latini, 2015; Latini et al., 2015b).

Before the start of dissection, the superficial anatomy of the sulci and gyri was studied in detail. The specimens were dissected in a stepwise manner, from lateral surface to the medial structures with a modified Klingler’s technique (Agrawal et al., 2011; Latini et al., 2015a, b, 2017). Microscopic metal dissectors and thin wooden spatulas were used in the initial steps of the dissection to split or partially peel away the brain cortex, preserving the most superficial intra-cortical and subcortical fibers of the lateral and basal brain surfaces. The dissections were performed by two neurosurgeons trained on white matter dissection (FL and GT) under microscopic magnification up to 10x (Zeiss OPMI Neuro NC-4, Carl Zeiss AG, Germany). Between each dissection session the specimens were placed in 5% formalin.

### Tractography and Virtual Dissection of the MdLF

#### Participants

Twenty-four subjects (14 females and 10 males; age groups 26–30, 31–35, and 36+) were acquired from the Human Connectome Project (HCP) database. The subjects were selected because of the older age group, high quality structural images including T1 and T2 sequence and high angular resolution diffusion imaging (dMRI). The 1200 Subject release Diffusion data acquisition and preprocessing are included in the 1200 Subject release and are summarized below. The 1200 Subjects Data Release Reference Manual provides full technical documentation and can be found freely downloadable at https://db.humanconnectome.org.

#### Image Acquisition and Reconstruction

HCP data were acquired using a Siemens Skyra 3.0 T with a 32-channel head coil (Siemens Healthineers, Erlangen, Germany) according to the HCP Study Protocol (Van Essen et al., 2013). T1w image were acquired using the 3D MPRAGE sequence with 0.7 mm isotropic resolution (FOV = 224 mm, matrix = 320, 256 sagittal slices in a single slab), TR = 2400 ms, TE = 2.14 ms, TI = 1000 ms (Glasser et al., 2013). Diffusion data was acquired with a multi-shell diffusion scheme (*b*-values 1000, 2000, and 3000 s/mm^2^), each shell with 90 diffusion sampling directions. The in-plane resolution and slice thickness were 1.25 mm, with TR = 5500 ms, TE = 89 ms, flip angle = 78 degrees using a multiband factor of 3.

All HCP diffusion datasets were preprocessed using the HCP MR Diffusion Pipeline (v3.19.0) which includes; EPI distortion correction using FSLs TOPUP algorithm, eddy current and motion correction using FSLs EDDY algorithm, gradient non-linearity correction and calculation of *b*-value/*b*-vector deviation (Andersson et al., 2003; Sotiropoulos et al., 2013; Andersson and Sotiropoulos, 2016). HCP diffusion data were reconstructed in DSI Studio^1^ using the generalized *q*-sampling imaging approach (Yeh et al., 2010) with a diffusion distance ratio of 1.2.

Prior to reconstruction, all included datasets were thoroughly examined to ensure the quality and integrity of diffusion data using the built-in quality control in DSI studio and by visual examination (Yeh et al., 2019).

#### Fiber Tracking and Analysis

We performed deterministic fiber tracking using DSI Studio software (DSI Studio^2^), which utilizes a generalized streamline fiber tracking method (Yeh et al., 2013). Parameters selected for fiber tracking included a step size of 0.2 mm, a minimum fiber length of 20 mm, and a turning angle threshold of 45°. For progression locations containing > 1 fiber orientation, the fiber orientation most congruent with the incoming direction and turning angle < 45° was selected to determine subsequent moving direction. Each progressive voxels’ moving directional estimate was weighted by 20% of the previous voxels incoming direction and by 80% of its nearest fiber orientation. This sequence was repeated to create fiber tracts. Termination of the tracking algorithm occurred when the quantitative anisotropy (QA) (Yeh et al., 2013) dropped below a subject-specific value: when fiber tract continuity no longer met the progression criteria or when 100,000 tracts were generated. We pre-selected QA termination threshold, between 0.02 and 0.08, by analyzing the number of false continuities generated within each subjects’ dataset and choosing the compromise value that allowed optimal anatomical detail with minimal noise. In the same way, we selected a smoothing parameter of 50%, a process also been described by other authors (Panesar et al., 2018).

#### Region of Interest Placement and Fiber Selection

**FIGURE 1:**
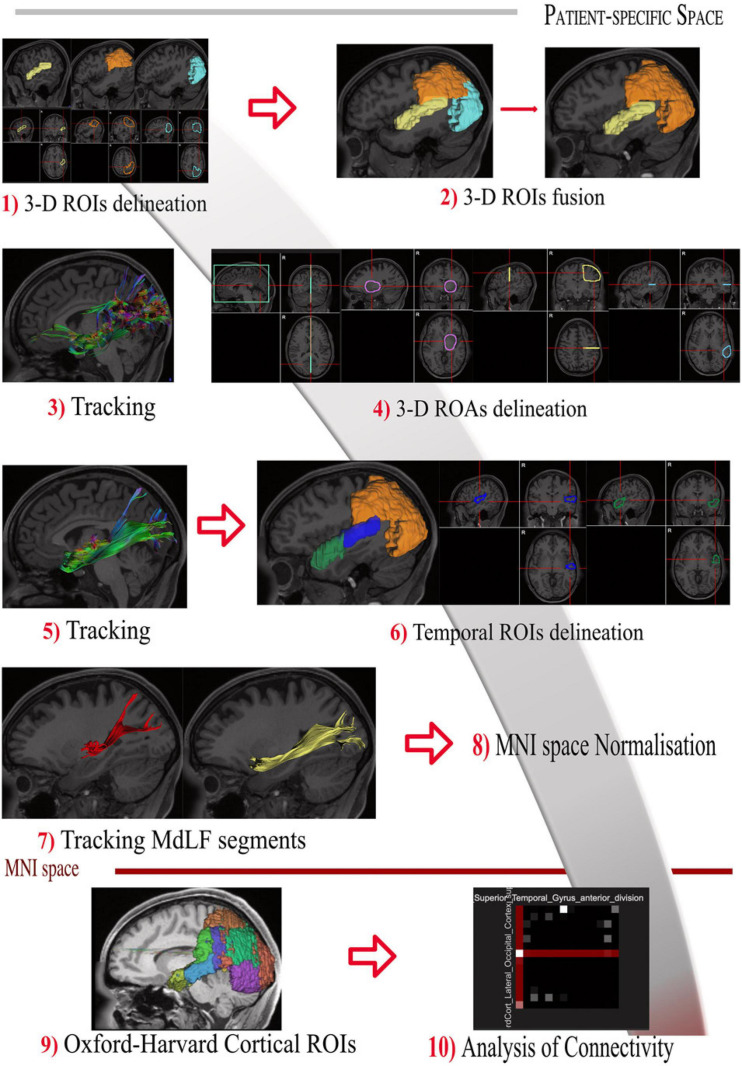
Tractography protocol for the identification and segmentation of MdLF in 10 steps. **(Step 1)** The 3-D ROIs are drawn delineating the entire STG (yellow), parietal lobe (orange) and occipital lobe (light blue) displayed with 3D reconstructions and 3-axis projection. **(Step 2)** Parietal and occipital lobe are merged in one ROI to delineate the entire temporo-parieto-occipital connectivity. **(Step 3)** The whole cortical subcortical connectivity of the temporo-parieto-occipital region was then revealed, displayed here in sagittal projection and with directional colors. **(Step 4)** The 3D orientation allows us to identify MdLF fibers in their central longitudinal portion (stem), as well as other close white matter tracts. Fibers crossing or close to the midline were identified and subtracted with the green region of avoidance (ROA). The IFOF fibers were identified as medial and crossing the external capsule and excluded with the purple spheroidal ROA; Fibers of the acoustic radiation were found to be medial and perpendicular to the MdLF stem fibers at the level of the aTTG. They were also excluded if present with the posterior portion of the purple spheroidal ROA including the postero-lateral portion of the thalamus and basal ganglia. Fibers from the arcuate fasciculus (AF) were identified as lateral and almost perpendicular with respect to the MdLF stem fibers; the horizontal component of the SLF was lateral and dorsal in respect to the MdLF stem fibers. Both these two bundles were excluded with the Yellow ROA placed at the level of the central sulcus. The vertical portion of the SLF was identified as perpendicular, lateral and posterior in respect to the MdLF stem and excluded with the light blue ROA. In case ILF fibers were identified ventral and lateral in respect to the MdLF stem fibers the light blue ROA was extended to the MTG and ITG. **(Step 5)** Once the whole MdLF course was isolated, each MdLF temporal termination was identified using an additional one-ROI approach in each cortical/subcortical territory. **(Step 6)** The temporal region was then divided into two sub-regions to reveal the two components of MdLF. The posterior temporal region (blue) included the posterior portion of the superior temporal gyrus, aTTG, and PT. The anterior temporal region (green) included the anterior STG (aSTG), the planum polare (PP), and the superior part of the TP. **(Step 7)** The temporal ROIS were used to track the anterior component of MdLF (aMdLF, yellow) and the posterior segment of MdLF (pMdLF, red). **(Step 8)** The Harvard-Oxford Cortical Atlas was spatially normalized to subject-specific space for each subject using the built-in normalization routine in DSI Studio. **(Step 9)** Regions involved in the MdLF pathway were included and modified to account for normalization errors, anatomical differences, and fiber tracking artifacts, based on *a priori* knowledge gained from the dissection. **(Step 10)** Connectivity matrices were generated by the Connectivity Matrix function in DSI Studio for each segment (including left and right MdLF, pMdLF, and aMdLF).

DSI Studio was used to place and draw regions of interest (ROIs) and anatomical landmarks. The manual ROIs were placed on T1-weighted maps with locations according to the anatomical findings in the white matter dissection. The visual steps to identify and segment the MdLF and its subsegments are displayed in Figure 1. Shortly, one 3-dimensional ROI included the entire occipital lobe from the posterior portion of the POS medially projecting laterally toward to the temporo-occipital junction. The anterior margin of the occipital lobe was defined by the line connecting the inferior parietal lobe, angular gyrus and temporo-occipital junction (preoccipital notch). The second ROI included the whole parietal lobe ipsilaterally from the POS medially, the postcentral gyrus anteriorly, and the IPL and angular gyrus laterally. The whole STG (including the medial anterior transverse temporal gyrus, aTTG) defined posteriorly by the parietal part of the supramarginal gyrus, was used as the third ROI (Figure 1, steps 1–2). The whole cortical subcortical connectivity of the temporo-parieto-occipital region was then revealed (Figure 1, step 3) to virtually dissect the MdLF. Once the MdLF fibers were identified in their central longitudinal portion (stem) lateral, posterior and slightly inferior compared with the posterior insular cortex, the other close white matter tracts were identified and subtracted with selected region of avoidance (ROAs) (Figure 1, step 4). Fibers from the arcuate fasciculus (AF) were identified as lateral and almost perpendicular with respect to the MdLF stem fibers; the two indirect (vertical and horizontal) components of the SLF were both lateral and respectively posterior and dorsal in respect to the MdLF stem fibers. The inferior fronto-occipital fascicle (IFOF) fibers were identified as medial and crossing the external capsule; the inferior longitudinal fascicle (ILF) fibers were ventral and lateral with respect to the MdLF stem fibers. Fibers of the acoustic radiation were found to be medial and perpendicular to the MdLF stem fibers at the level of the aTTG. Once the whole MdLF course was isolated (Figure 1, step 5), each MdLF temporal termination was identified using an additional one-ROI approach in each cortical/subcortical territory (Figure 1, step 6). Based on the anatomical findings in the cadaver dissection, the temporal region was then divided into two sub-regions to reveal the two components of MdLF. The lateral surface of the STG was segmented in an anterior and a posterior portion using a virtual lateral projection of the limit on the dorsal surface between the planum polare (PP) and the aTTG. This landmark was also in agreement with the anterior and posterior subdivision of the superior temporal gyrus provided by Harvard-Oxford Cortical Atlas distributed by DSI studio which was adopted for the streamline analysis (described below). Therefore, the posterior temporal ipsilateral region (pT-ROI) included the posterior portion of the superior temporal gyrus, aTTG, and PT was used to track the posterior segment of MdLF (pMdLF); the anterior temporal ipsilateral region (aT-ROI) included the anterior STG (aSTG), the PP, and the superior part of the TP (Figure 1, step 7) was used to track the anterior component of MdLF (aMdLF).

#### Streamline Analysis

The Harvard-Oxford Cortical Atlas (Desikan et al., 2006; Schurz et al., 2017) was spatially normalized to subject-specific space for each subject using the built-in normalization routine in DSI Studio (Figure 1, steps 8–9). Regions involved in the MdLF pathway were included and modified to account for normalization errors, anatomical differences, and fiber tracking artifacts, based on *a priori* knowledge gained from the dissection. Connectivity matrices were generated by the Connectivity Matrix function in DSI Studio for each segment (including left and right MdLF, pMdLF, and aMdLF) (Figure 1, step 10). In total, six connectivity matrices were generated per subject, to give a total of 144 matrices over all subjects. A connectivity matrix provides the number of reconstructed streamlines between two given regions (as defined above); these streamline counts were divided with the total amount of streamlines for the given MdLF component and presented as percentage (referred to as normalized streamline counts). Average normalized streamline counts and corresponding standard deviation was calculated. Upscaled cumulative values were used to generate connectograms (circle graphs) for each MdLF component using CIRCOS^3^. Average normalized streamline counts not reaching 3.0% and connections between unrelated regions were excluded.

### Statistical Analysis

For descriptive analysis, means and standard deviations were calculated for each tract metric for all tracts and visualized through box plots including mean value, 25th and 75th percentile, and min to max. Mann–Whitney *U*-test for independent samples was used for comparison between groups for tract metrics (volume, length, FA, mean diffusivity, MD, axial diffusivity, AD and radial diffusivity, RD) for all MdLF components. The symmetry coefficient of each tract metric and MdLF component was calculated, based upon the formula (L – R)/(L + R) as have been reported in previous studies (Sreedharan et al., 2015; Latini et al., 2017). A *p*-value less than 0.05 was considered statistically significant.

The statistical package SPSS 25.0 (SPSS, Inc., Chicago, IL, United States) was used for the statistical analysis.

### Clinical Cases

To further discuss the MdLF functional role we selected two clinical cases where STG was involved by a low-grade glioma that could illustrate a lesion model of MdLF. Pre- and post-operative clinical, neuropsychological and neuroradiological data of the two cases are provided. These clinical cases were retrospectively retrieved by a database containing clinical and radiological data of patients with suspected low-grade gliomas operated on at the Department of Neurosurgery, Uppsala University-Hospital, Uppsala, Sweden, who had detailed pre- and postoperative neuropsychological and speech evaluation as a part of a larger research project. The regional ethics committee, Regionala Etikprövningsnämnden Uppsala, approved the study protocol (Dnr 2015/210). An informed consent from patients included in this study was acquired.

## Results

### Anatomical Dissection

**FIGURE 2:**
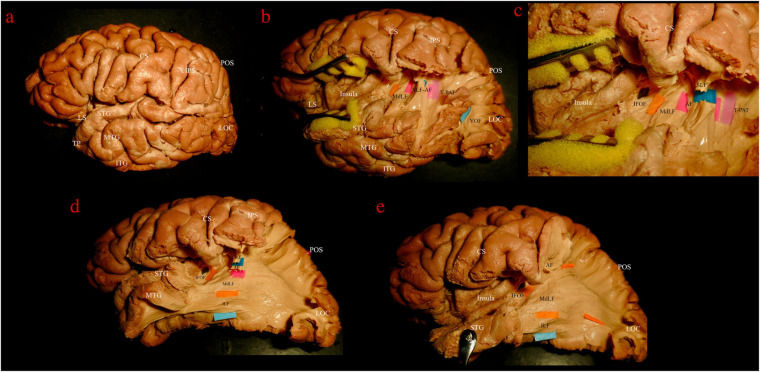
Left hemisphere, lateral view. Stepwise dissection from lateral to medial structures. **(a)** Intact hemisphere. Relevant gyri and sulci for present dissection and orientation are tagged: central and lateral sulci (CS, LS); temporal pole (TP) and superior, middle and inferior temporal gyri (STG, MTG, and ITG); intraparietal sulcus (IPS); parieto-occipital sulcus (POS); lateral occipital cortex (LOC). **(b)** Dissection of temporo-parietal-occipital junction (TPOJ) area. From lateral to mesial, after removing U-fibers, different layers of associative bundles are encountered. Superficial layer: vertical component of superior longitudinal fascicle (vSLF, dark blue tag) and vertical occipital fascicle (VOF, light blue tag). Upper intermediate layer: arcuate fascicle (AF, magenta tag) and temporo-parietal aslant tract (T-PAT, pink tag). Lower intermediate layer: middle longitudinal fascicle (MdLF, orange tag). **(c)** Detailed view of the white matter layers at the deep TPOJ. vSLF has been interrupted to show the underling AF. Lower layer, formed by the inferior fronto-occipital fascicle (IFOF, black tag) is now shown as running more medial through the posterior sub-insular region and external capsule. **(d)** VOF, T-PAT and the inferior, temporal, aspect of vSLF-AF have been removed uncovering the inferior longitudinal fascicle (ILF, light blue tag), which runs inferior and parallel to the MdLF fibers within the SS and the temporal lobe. **(e)** The STG has been reflected and SLF and AF further removed in order to show the course of MdLF from STG to the parieto-occipital cortex.

The dissection started from the lateral surface of the hemisphere (Figure 2a) with the exposure of the indirect vertical component of the superior longitudinal fasciculus (vSLF) that connects the AG to the region of the temporo-parietal-occipital junction (Figure 2b). Posterior and on the same plane of the vSLF, the vertical-occipital fascicle (VOF) is encountered. The direct component of the SLF, the AF, with its typical C-shaped perisilvian course between the infero-lateral temporo-occipital region and the inferior frontal gyrus, was then detected mesial and slightly anterior to vSLF (Figures 2b,c). After removing the intermediate U-fibers of the posterior temporo-parietal junction the temporo-parietal aslant tract (T-PAT) was identified at the same level of the AF (Figure 2b) (Panesar et al., 2019).

**FIGURE 3:**
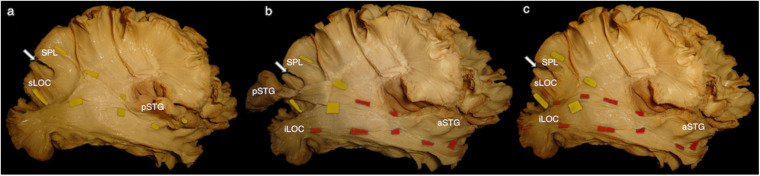
Right hemisphere, infero-lateral view. **(a)** Posterior middle longitudinal fascicle (pMdLF). Yellow tags underline the path of the pMdLF, which is the posterior, superficial branch of the MdLF: anterior terminations are in the posterior superior temporal gyrus (pSTG), namely anterior temporal transverse gyrus and the planum temporale; posterior terminations go across the parieto-occipital sulcus (POS; white arrow) at the superior parietal lobule (SPL) and at the superior lateral occipital cortex (sLOC). **(b)** Anterior middle longitudinal fascicle (aMdLF). pSTG has been disconnected and the stripped posteriorly along with pMdLF fibers, showing the deeper, anterior segment of the MdLF (aMdLF). Red tags show the path of the aMdLF from the anterior superior temporal gyrus (aSTG), including planum polare and temporal pole, to the LOC. **(c)** After completely removing pSTG and main fibers of the pMdLF, the entire aMdLF is uncovered. While no fibers of the aMdLF were detected above the POS, we found that both the aMdLF and pMdLF had terminations at the level of the sLOC. Only the aMdLF showed cortical terminations at iLOC.

**FIGURE 4:**
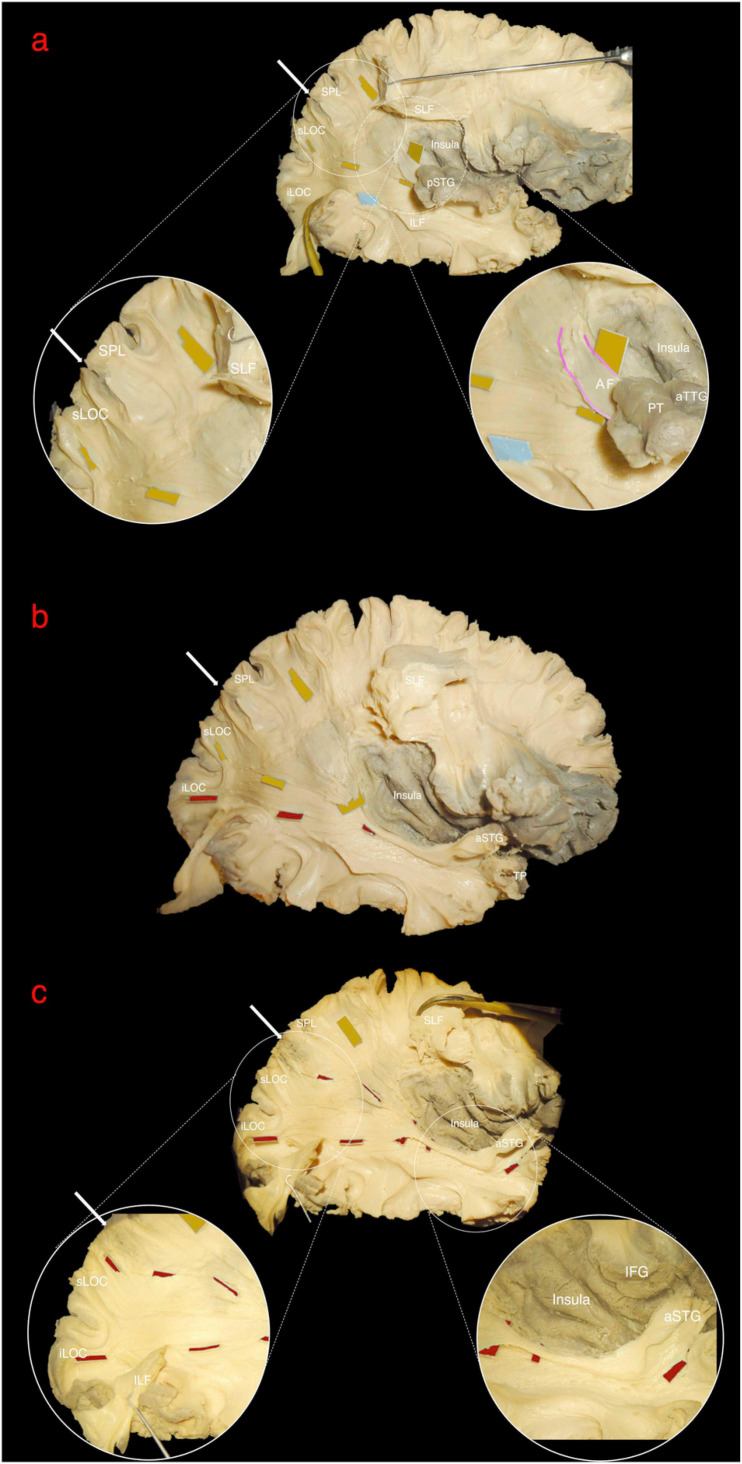
Right hemisphere, lateral view. The white arrow indicates the parieto-occipital sulcus (POS). **(a)** Superior longitudinal fascicle (SLF) has been sectioned and lifted to uncover the posterior middle longitudinal fascicle (pMdLF, yellow tags) fibers running from posterior superior temporal gyrus (pSTG) to superior parietal lobule (SPL) and superior lateral occipital cortex (sLOC). The inferior longitudinal fascicle (light blue tag) running from temporal pole to inferior lateral occipital cortex (iLOC) is also shown and partially retracted downward. Circles show a magnification of the anterior and posterior terminations of the pMdLF. Anteriorly, a small amount of fibers from planum temporale (PT) and anterior temporal transverse gyrus (aTTG), i.e., the pSTG, join the direct component of the SLF (arcuate fasciculus – AF, pink gradient lines). These fibers are more superficial than pMdLF fibers, which have a more posteriorly oriented direction. **(b)** vSLF and part of the anterior portion of pMdLF have been removed to show the relationship between the two segments of the MdLF: the pMdLF runs more superficially and cranially than the anterior MdLF (aMdLF, red tags). **(c)** The pMdLF has been completely removed and the posterior terminations of ILF displaced inferiorly, showing the entire course of the aMdLF from the anterior STG (aSTG) to the sLOC and iLOC. Circles show the magnification of the anterior and posterior terminations of the aMdLF.

The central segment of the MdLF lies underneath the AF at the level of the STG (Figures 2b–d). Its fibers run in an antero-posterior direction in the deep white matter of the STG directed toward the parieto-occipital junction. Posteriorly, these fibers fan to cover a broad area going from the SPL and PreCu to the Cu and LOC (Figures 2e, 3). These posterior terminations are partially covered by the posterior terminations of the ILF, which runs inferior and parallel to the MdLF fibers within the temporal lobe and SS (Figures 2d,e) and slightly more lateral to MdLF fibers at the level of the occipital lobe (Figure 4c). Following the bundle anteriorly, we consistently documented a superficial, posterior branch (pMdLF) terminating at the posterior superior temporal gyrus, namely at the aTTG and PT, and a deeper, anterior branch (aMdLF) terminating at the PP, lateral aSTG, superior temporal sulcus (STS), and TP. Delicate dissection of the fibers of these two subcomponents showed that the anterior, deeper, branch of the MdLF (aMdLF) runs from the PP, lateral aSTG, and TP to the LOC (both superior gyrus, sLOC, and inferior gyrus, iLOC, described by their relationship with the lateral occipital sulcus), and to lesser extent to the lateral extension of the Cu. The posterior, superficial, branch of the MdLF (pMdLF) has its anterior terminations in the aTTG and PT and its posterior terminations across the parieto-occipital sulcus (POS) at the SPL and the lateral extension of the PreCu and Cu. These subcomponents were detected in all dissected hemispheres (Figures 3a–c, 4a–c).

### Spatial Relationship With Adjacent Fiber Bundles

The MdLF is part of the sagittal stratum (SS) described by Sachs, a deep white matter structure of the temporo-parietal occipital region, organized in three layers: fibers of the splenium (forceps corporis callosi), optic radiations (OR) (stratum sagittalis internum) and the association pathways (stratum sagittalis externum, including IFOF, MdLF, and ILF) (Forkel et al., 2015). Since in its classical classification the external layer only included ILF as a main component, it is worth mentioning that IFOF and MdLF are integral components of the external layer of SS with even larger fibers contribution to the layer compared with ILF.

Both fiber dissection and fiber tracking confirmed an intimate anatomical relation of the MdLF with the AF/SLF, ILF, IFOF, and ORs.

Vertical SLF and AF are more superficial and perpendicularly intersect the MdLF at the level of the temporo-parietal junction (Figures 2b,c). VOF, located at the temporo-occipital junction, lies on the same plane of vSLF and intersects perpendicularly the fibers running from the temporal to parietal and occipital lobes (Figure 2b). In a deeper layer, ILF runs parallel, more caudal and superficial to the MdLF, partially covering its occipital (LOC and Cu) terminations (Figures 4a–c).

The IFOF is part of the external capsule and crosses from the frontal to the temporal lobe at the level of the limen insulae. Its fibers run on the roof of the temporal horn of the lateral ventricle in a deeper layer in respect to the MdLF fibers. The two bundles form an angle of about 40° at this level (Figure 2c). Posteriorly, white matter tracts are extremely packed in the layered, onion-like, structure of the SS, with cortical terminations of the MdLF being more superficial but mostly overlapping with the cortical terminations of the IFOF. To avoid contamination of IFOF fibers during the MdLF dissection, the aMdLF fibers were dissected as a single layer from the anterior portion of STG to the occipital lobe through the external layer of SS. The insular cortex and the external capsule were preserved in our dissection to assure that the IFOF fibers were more intact medially when spatially compared with the MdLF. More deeply, ORs fibers directed to the Cu and lingual gyrus were encountered.

Within the superior temporal gyrus, the pMdLF shows close relationship with the superficial fibers of the AF laterally, and with the acoustic radiation which is located medial and caudal, underneath the aTTG (not displayed in our figures). The aMdLF runs instead parallel to the ILF within the temporal region until their division in the anterior and caudal part of the superior temporal gyrus and sulcus (Figures 2, 4) as previously reported (Latini et al., 2017).

### Diffusion Tensor Tractography (DTT) Segmentation of the MdLF Subcomponents

**FIGURE 5:**
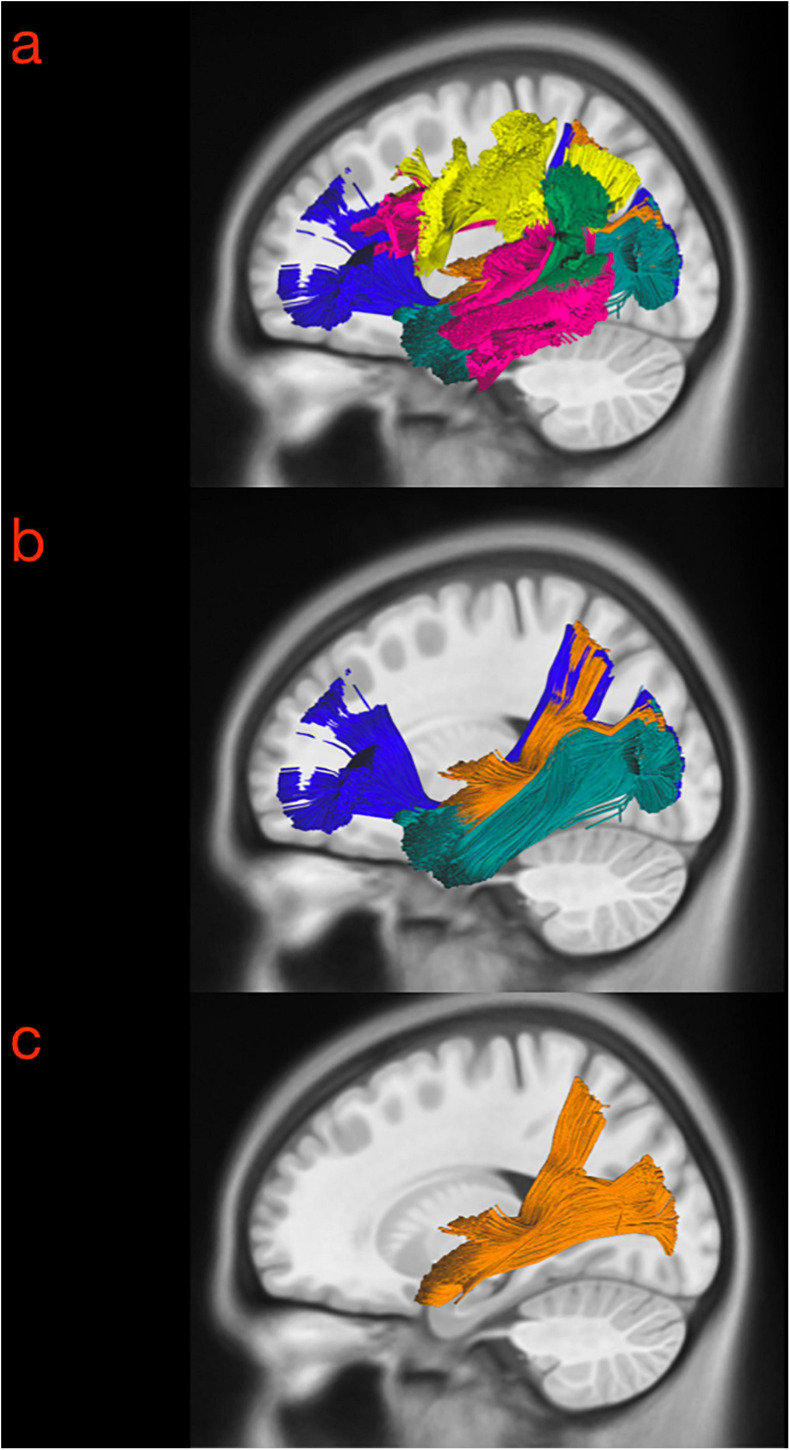
Three exemplary steps of the virtual in-vivo dissection technique. **(a)** The first and more lateral white matter bundle such as the vSLF (green), hSLF (yellow) were identified as well as the course of the AF (pink), which is lateral and posterior compared to the MdLF in its temporal component, and represented the landmark for the identification of the MdLF stem. **(b)** ROAs were then subtracted from the ROIs describing the AF and vSLF to show the superficial layer of the sagittal stratum including MdLF fibers (orange) and ILF fibers (dark green). **(c)** The ILF, IFOF (dark blue), optic radiation (light blue) medial to this level were subtracted from the virtual dissection according to the clear anatomical differences in the course of their fibers. As a result, the MdLF stem was identified and isolated in three-dimensional space.

**FIGURE 6:**
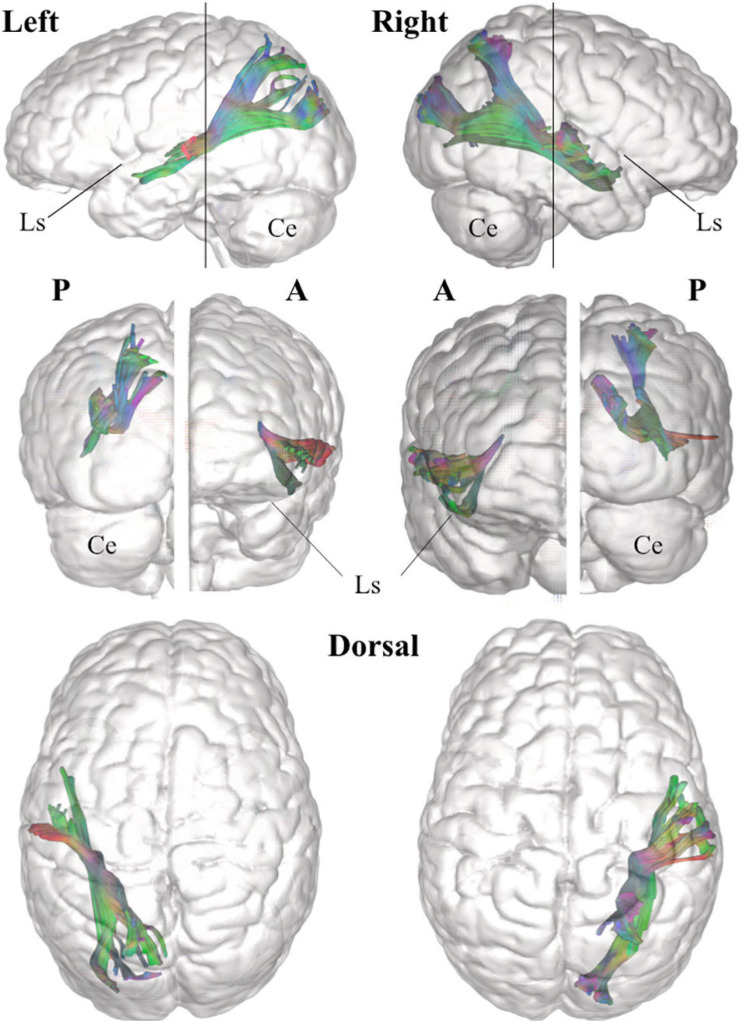
Three-dimensional reconstruction within a “glass-brain” of origin, course, and terminations of the MdLF (complete) of the left and right side on sagittal projection **(first row)**, on coronal projection (from both posterior and anterior view, **second row**) and axial dorsal view **(third row)**. The color of the white matter pathway has been maintained directional to show details about the temporal and parieto-occipital terminations, which clearly bend from the stem of the pathway. A, anterior view; P, posterior view; Ls, lateral sulcus; Ce, cerebellum.

**FIGURE 7:**
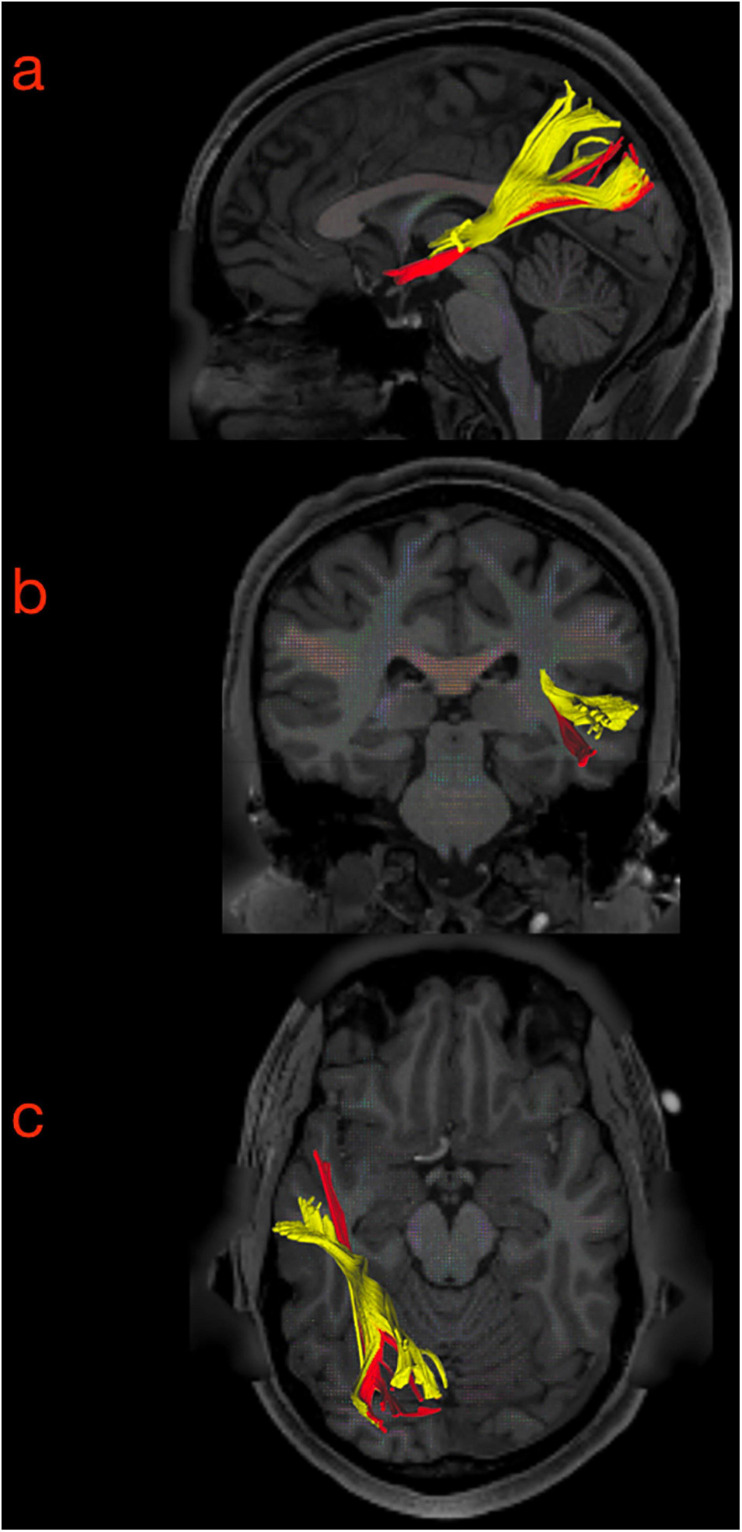
Sagittal **(a)**, coronal **(b),** and axial **(c)** slice of the tractography reconstruction of the two MdLF components on the left-sided hemisphere (axial slice in neurological projection). In the temporal region, the subcomponents were clearly distinguished from the stem to their terminations. The anterior component (originating from the anterior portion of the superior temporal gyrus, aSTG, and planum polare, PP) was named the aMdLF (red). The posterior portion (originating from the posterior portion of superior temporal gyrus, pSTG, anterior temporal transverse gyrus, aTTG, and planum temporale, PT) was named the pMdLF (yellow).

**FIGURE 8:**
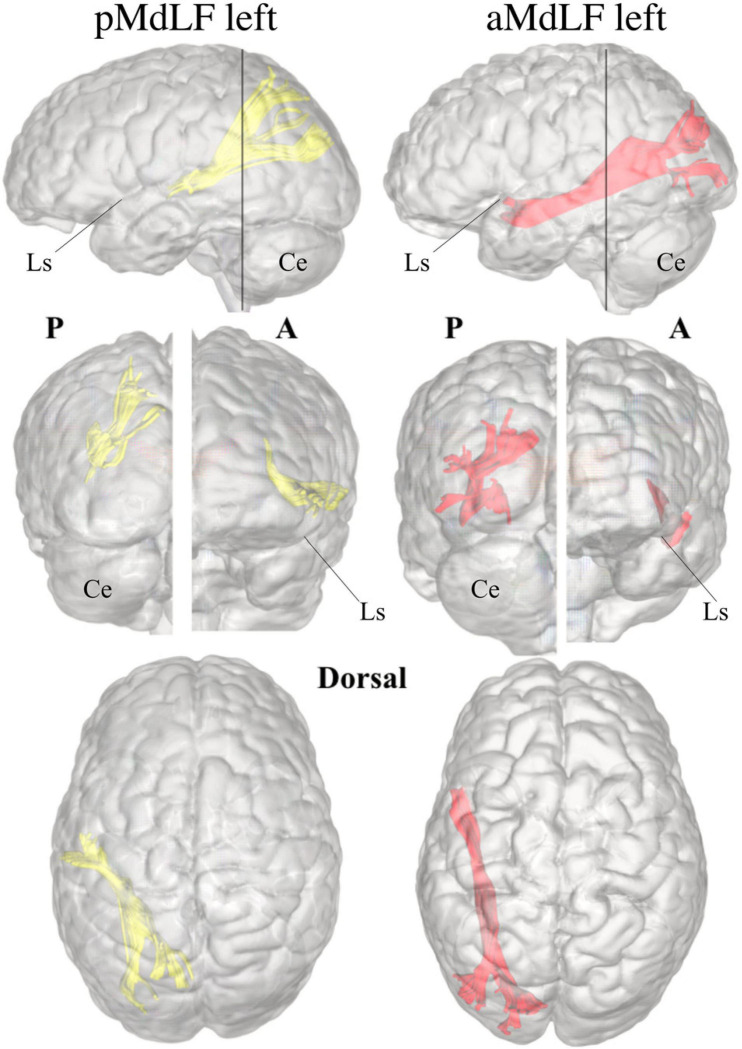
Three-dimensional reconstruction within a “glass-brain” of origin, course, and terminations of the two MdLF segments, displayed separately. The pMdLF (yellow) and aMdLF (red) are displayed on the left side on sagittal projection **(first row)**, on coronal projection (from both posterior and anterior view, **second row**), and axial dorsal view **(third row)**. See the text for further details. A, anterior view; P, posterior view; Ls, lateral sulcus; Ce, cerebellum.

The technique we used for virtual dissection reproduced our anatomical findings in the white matter dissection, and following the same step-by-step anatomical criteria, the MdLF was successfully isolated in all subjects. The first and more lateral white matter bundles like the vSLF were subtracted, showing the course of the AF, which is lateral and posterior compared to the MdLF in its temporal component, and represented the landmark for the identification of the MdLF stem within superficial layer of the sagittal stratum together with the ILF fibers (Figures 5a,b). The ILF, IFOF, and OR medial to this level were subtracted from the virtual dissection according to the clear anatomical differences in the course of their fibers. As a result, the MdLF stem was identified and isolated in three-dimensional space (Figure 5c). The origins, course, and terminations of this bundle are shown in Figure 6. In the temporal region, the subcomponents were clearly distinguished from the stem to their terminations (Figures 7a–c). Both the aMdLF and the pMdLF were consistently found, bilaterally in all the subjects. The origins, course, and terminations of the two branches are shown in Figure 8 and Supplementary Materials 1, 2.

The whole MdLF did not show quantitative differences in terms of volume, length or tract metrics between left and right hemisphere. The aMdLF and pMdLF displayed similar volume, length or tract metrics without any significant difference in lateralization. Tract metrics were analyzed to exclude differences between sub segments which might have resulted in artifacts especially in regions like the sagittal stratum (periventricular portion of the temporo-parieto-occipital intersection area) where many kissing and crossing fibers can be tracked. A summary of descriptive and statistical results is displayed in Table 1.

**TABLE 1:** Summary of descriptive results at group level with means, standard deviations, (SD) and range for all the tracts indices (upper part with volume, tract length, tract FA-value; lower part with MD, AD, and RD) for all subgroups.

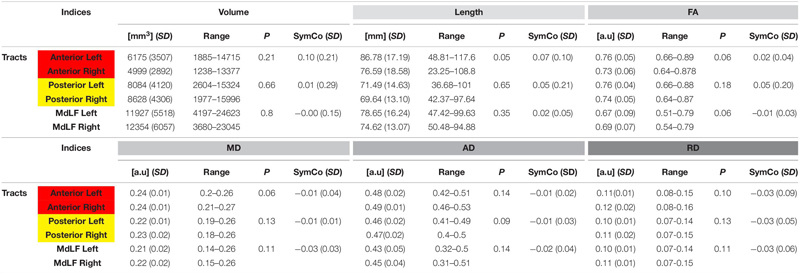

*Mann–Whitney *U*-test for independent samples was used for comparison between groups for volume, length, FA, MD, AD, and RD. A *p*-value less than 0.05 was considered statistically significant. SymCo: symmetry coefficient, from the formula (Left – Right)/(Left + Right). A positive value reflects lateralisation to the left side, while a negative value reflects lateralisation to the right; Zero (0) reflects that both sides were equal in the measured value. FA. fractional anisotropy; MD, mean diffusivity; AD, axial diffusivity; RD, radial diffusivity; a.u, absolute unit. The yellow and red colors consistently reflect the color code used for the MdLF subsegments as displayed by white matter dissection and tractography results.*

### Analysis of MdLF Sub-Segments Connectivity

**FIGURE 9:**
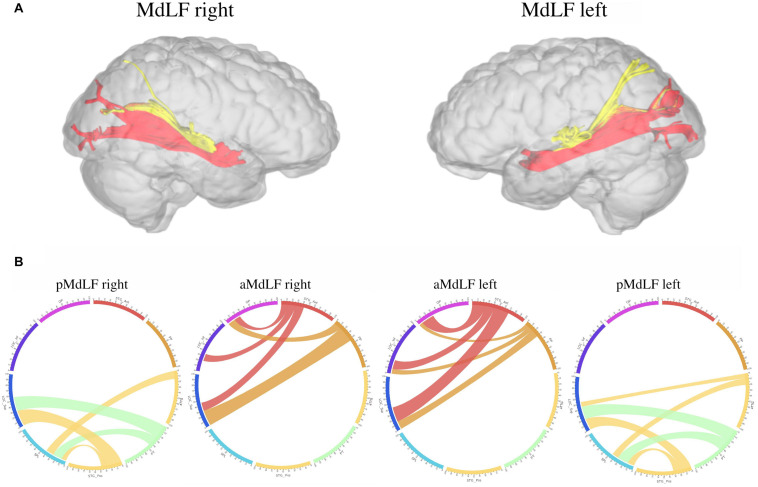
Connectivity analysis at group level for the two subcomponents of the MdLF on both sides, with three-dimensional reconstruction of the illustrative pathways **(A)** and a circle diagrams to show the connectivity between the selected regions from the Harvard-Oxford Cortical Atlas **(B)**. The number associated to the cortical areas define the normalized number of streamlines (percentage) connecting the two regions with a cut-off of 3%. The aMdLF (red) was found to connect the anterior portion of the superior temporal gyrus (aSTG) and the PP on both sides with the occipital region only. Within the occipital region, three areas where consistently connected by the aMdLF: the superior portion of lateral occipital cortex (sLOC), which received 40% of the fibers on the left side and 45% on the right side; the inferior portion of lateral occipital cortex (iLOC), with 25% on the left side and 10% on right side; and the occipital pole (OP) receiving 25% of the fibers on the right side and 32% on the left side. The pMdLF (yellow) displayed a constant connection between the posterior portion of the superior temporal gyrus, the anterior temporal transverse gyrus (aTTG) and planum temporale (PT) with the parieto-occipital region. Fibers from the aTTG were found in connection with the superior parietal lobe (SPL) bilaterally (10% on the left side and 15% on the right side) and with sLOC on the left side only (10%). The PT displayed a connection with the SPL bilaterally (20% on the left side, 15% on the right side) and with the sLOC (25% on the right side and 22% on the left side). The posterior portion of the superior temporal gyrus (pSTG) displayed bilateral connections with the SPL (10% on the left side, 8% on the right side) and with sLOC (20% on the left side, 30% on the right side).

A connectivity analysis at group level for the two subcomponents of the MdLF on both sides was performed with three-dimensional reconstruction of the illustrative pathways. A circle diagram was chosen to show the connectivity between the selected regions from the Harvard-Oxford Cortical Atlas (Figure 9).

#### Anterior MdLF (aMdLF)

Analysis at the group level showed that the aMdLF connects the aSTG and the PP with the occipital region in both right and left hemisphere (Figure 9). Within the occipital region, three areas where consistently connected by the aMdLF: the superior portion of lateral occipital cortex (sLOC), the inferior portion of the lateral occipital cortex (iLOC), and the occipital pole (OP) on the both sides. A slight asymmetry, was detected on the left side with higher connectivity between the PP and the iLOC.

#### Posterior MdLF (pMdLF)

The pMdLF displayed a constant connection between the posterior portion of the superior temporal gyrus (pSTG), the aTTG and PT and the parieto-occipital region (Figure 9). The most important connections were found between the aTTG and SPL bilaterally, between the PT and SPL bilaterally, and between the PT and sLOC.

### Clinical Cases

#### Case #1: aMdLF

**FIGURE 10:**
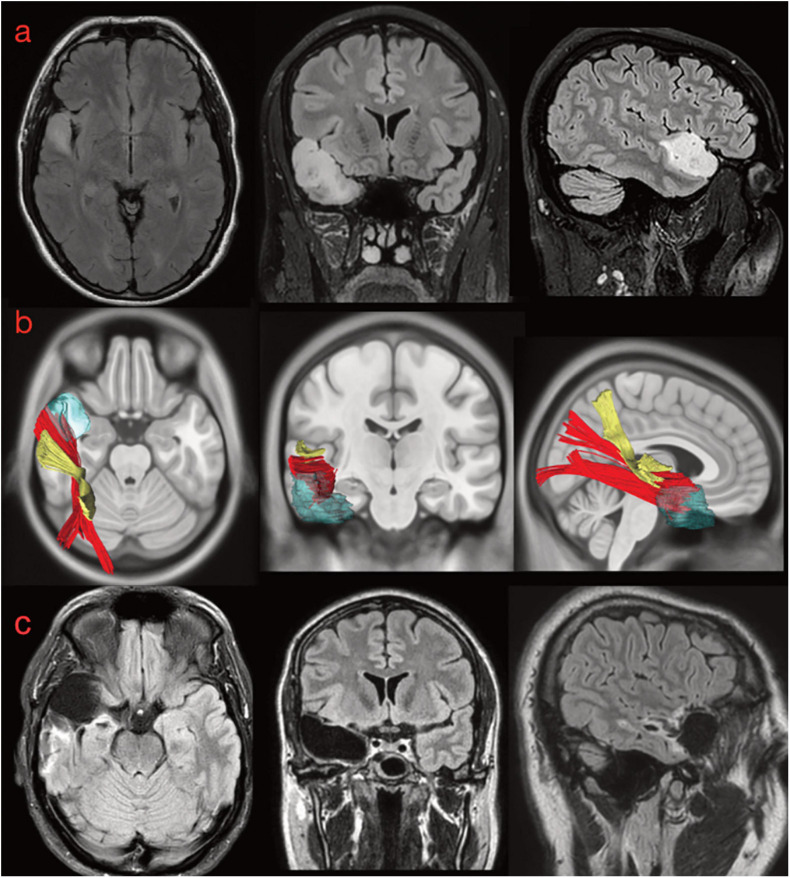
**(a)** Preoperative morphological MRI T2-Flair sequences of illustrative case #1. The hyperintensity signal on axial, coronal, and sagittal (first row) views show a suspected low-grade glioma involving the anterior and superior portion of the superior temporal gyrus on the right side. The tumor invaded even the medial component of the temporal pole (on the coronal). **(b)** The normalization of the tumor lesion volume within the MNI space allowed us to merge our anatomical results on the MdLF dissection with the patient-specific anatomy. According to our results, the tumor invaded the aMdLF (red) but not the pMdLF (yellow). **(c)** Postoperative MRI T2-Flair images showing a complete resection of the tumor lesion.

A 34-year-old patient was admitted to our clinic because of seizures in 2016. MRI investigation detected a suspected low-grade glioma in the aSTG on the right side (Figure 10a). Seizure is described as a form of focal epilepsy with déjà vu phenomena and auditory hallucinations (melodies) evoked by other sounds. The preoperative neuropsychological examination revealed impaired visuo-constructional ability but no deficits in learning and memory (see Supplementary Material 3 for details). A complete surgical resection of FLAIR-signal hyperintensity was performed (Figure 10c). The histopathological examination revealed an oligodendroglioma WHO II, and because of the radical resection, the patient was monitored with only serial MRIs. At the postoperative neuropsychological examination 3 months after the operation, the patient reported several psychiatric symptoms including obsessions (thoughts of self-harm) as well as compulsive behavior. The compulsions included counting and finger tapping of rhythms or melodies. According to the patient, these symptoms were markedly different from the déjà vu sensations and auditory hallucinations experienced preoperatively. The patient received antidepressant drugs with clear improvement of the psychological and psychiatric symptoms.

The retrospective analysis of white matter bundles involved by the tumor area and postoperative resection revealed that the aMdLF was mainly involved within the aSTG but even the most anterior portion of ILF was invaded in the TP (Figure 10b).

#### Case #2: pMdLF

**FIGURE 11:**
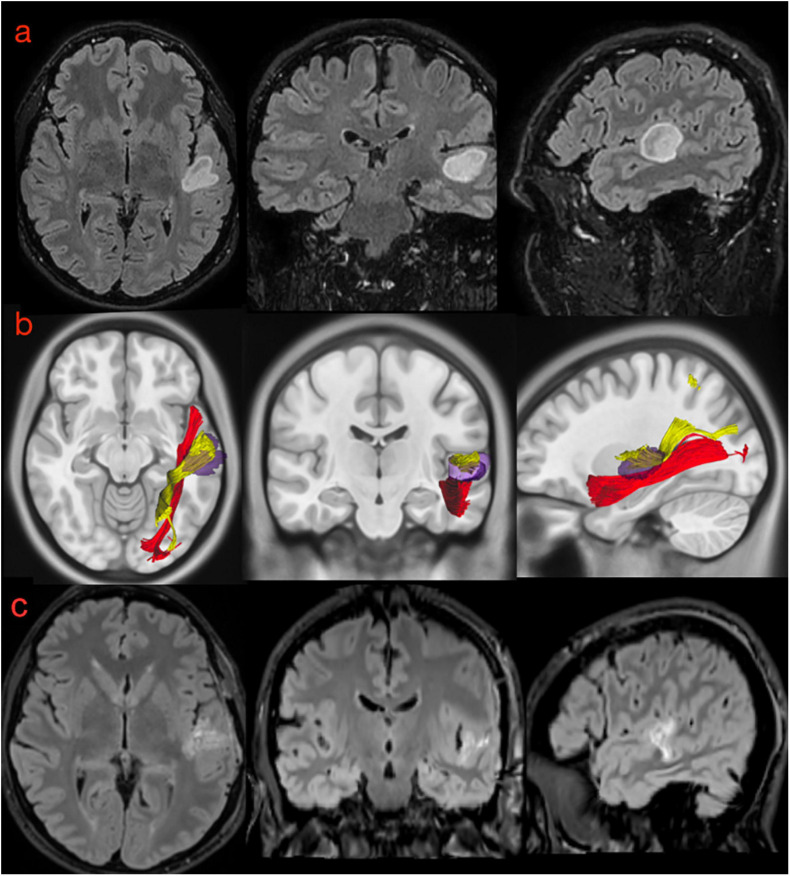
**(a)** Preoperative morphological MRI T2-Flair sequences of illustrative case #2. The hyperintensity signal on axial, coronal, and sagittal (first row) views show a suspected low-grade glioma involving the posterior portion of the superior temporal gyrus very close to anterior temporal transverse gyrus and the planum temporale. **(b)** The normalization of the tumor lesion volume within the MNI space allowed us to merge our anatomical results on the MdLF dissection with the patient-specific anatomy. According to our results, the tumor invaded the pMdLF (yellow) but not the aMdLF (red). **(c)** Postoperative MRI T2-Flair images showing a complete resection of the tumor lesion.

This 39-year-old patient was referred to our clinic because of cognitive impairment involving learning, memory, and concentration. An MRI investigation revealed an expansive lesion in the pSTG on the left side, without contrast enhancement (Figure 11a). At the neurological examination, the patient had no motor or sensory impairment; he described an intermittent whistling sound inside his head that made it difficult to perceive sounds in the surroundings. Speech examination revealed slight linguistic difficulties with affected expression ability, phonological word flow, and verbal working memory. He also started to complain some difficulty in comprehending verbal stimuli. Preoperative neuropsychological and language examination (see Supplementary Material 3 for details) confirmed the impaired learning and concentration in the audio-verbal domain. The patient refused surgery during awake monitoring and was therefore operated under general anesthesia with radical resection of the FLAIR-hyperintense signal (Figure 11c). The histo-pathological examination revealed an astrocytoma WHO II, IDH1 mutated, and the patient was treated with proton radiation therapy and adjuvant chemotherapy with temozolomide. Speech functions 3 months after the operation were impaired in all the domains, the relevant auditory contributions being: word mobilization, language understanding, and verbal working memory, while reading performance and spelling results improved. Neuropsychological examination was essentially unchanged compared with the preoperative results and showed impaired learning potential for verbal stimuli but not for visual stimuli. The analysis of white matter from the tumor region revealed that the pMdLF was the main structure affected by the astrocytoma, but that there was also minor infiltration of the short fibers of the AF on the left side (Figure 11b).

## Discussion

Despite being a long associative tract, MdLF has been object of debate since its first description. Indeed, different descriptions of MdLF cortical terminations, possible subdivisions and functional role have been reported in the scientific literature. Our work is placed in the context of such controversial debate and we tried to analyze each of the above aspects of the MdLF.

### Cortical Terminations of MdLF

The first controversy concerns MdLF posterior cortical terminations. Indeed, an associative bundle between TP/STG and IPL was initially described in monkeys (Seltzer and Pandya, 1984), and more recently in humans using *in vivo* virtual dissection, DTI-based, techniques (Makris et al., 2009). However, the absence of cortical terminations to the IPL (AG and/or SG) is probably the most striking dissonance between pure virtual dissection studies (Makris et al., 2009, 2013b, 2017; Menjot de Champfleur et al., 2013; Suzuki et al., 2018; Tremblay et al., 2019) and white matter dissection studies, including the present one. Indeed, our fiber dissection and high resolution fiber tracking study could not document the presence of MdLF terminations at IPL. This is consistent with previous anatomic studies showing that the MdLF connects the entire STG with the SPL and the occipital lobe (Maldonado et al., 2013; Wang et al., 2013; Kalyvas et al., 2020). This is probably due to the superior spatial resolution of white matter dissection compared to tractography analysis (Wang et al., 2008; Dell’acqua et al., 2010; Martino et al., 2013), which allows a clear documentation of the spatial relationship of the two branches of MdLF and of their cortical terminations, as shown in Figures 2–4.

Moreover, our results are supported by high resolution tractography, which allows direct investigation of the cortical termination of long-range fiber tracts and improved the connectomic analysis of white matter (Yeh et al., 2010, 2013; Wang et al., 2013; Panesar et al., 2018). However, both fiber microdissection and tractography analysis can be limited in detecting small superficial white matter fibers, which could limit the assessment of MdLF contribution to IPL (Martino et al., 2011; Menjot de Champfleur et al., 2013; Arnts et al., 2014; Reveley et al., 2015). Nonetheless, in this study we adopted a previously described modified fiber microdissection technique that was shown to have a high quality documentation of fine anatomical details as intra-cortical WM terminations (Latini et al., 2015b). Hence, in our study fibers terminating in the IPL from the STG were related to the vSLF and AF with no clear relationship with fibers of the MdLF indeed (Figure 4a). We agree with other authors suggesting that the previously described extension of the AF toward the anterior and middle segments of the superior temporal gyrus, as shown in multiple DTI studies with less spatial resolution (Catani et al., 2002; Fernández-Miranda et al., 2008), may represents an artifact due to the apposition of the MdLF and the arcuate fascicle at the temporoparietal junction (Maldonado et al., 2013; Wang et al., 2013).

### MdLF Subsegments and Organization

The second controversy about MdLF in the current literature concerns the possible organization of its fibers in 2 or more subcomponents. Indeed, tractographic studies showed up to six subcomponents of the MdLF (Makris et al., 2017), while the three available anatomic studies showed very contradictory findings as MdLF was showed as organized in a single bundle (Maldonado et al., 2013), in two components with a deeper layer of fibers originating more anteriorly and suggesting a segmentation pattern in the MdLF (Wang et al., 2013), and more recently in three subcomponents with different connection patterns (Kalyvas et al., 2020).

Our analysis consistently showed that MdLF is organized into two layers with different cortical terminations in 100% of specimens or subjects analyzed and in both the left and right hemispheres. The superficial branch of the MdLF connects the posterior regions of the STG, aTTG and PT with the SPL and sLOC and has been named the pMdLF. The deeper brunch, the aMdLF, primarily connects the aSTG/PP with the occipital lobe, with the iLOC, sLOC, and OP. A slightly higher connectivity was detected for the left MdLF subsegments. The left pMdLF displayed connections between aTTG primary with SPL but also with sLOC. The left aMdLF displayed a symmetrical occipito-temporal connection with both PP and aSTG connected with all three occipital regions (Figure 9). This leftward connectivity has not been reported by other authors but this is the first study to use quantitative registration of the connectivity between cortical regions connected by MdLF and therefore should be more carefully analyzed.

Despite these discrete interhemispheric differences in connectivity aMdLF and pMdLF showed a constant interhemispheric symmetry regarding volume length and tract metrics (Table 1). Tract metrics in our case are displayed to show interhemispheric symmetry and consistency between the tracts and therefore cannot be compared with results provided by other authors using different algorithms (Makris et al., 2013b, 2017; Menjot de Champfleur et al., 2013; Kalyvas et al., 2020). Our findings seem consistent with different techniques and therefore the aMdLF or pMdLF may not be considered artifacts based on DTI metrics and the intrinsic high angular resolution of imaging as described by other authors (Yeh et al., 2010, 2013).

Recently, Kalyvas et al. (2020) reported a segmentation in three layers of the MdLF, being the sub-segments related to aTTG (MdLF-I) and posterior transvers gyri (MdLF-II) more superficial than the sub-segment running from the most anterior part of the TP to the posterior border of the occipital lobe (MdLF-III). The latter sub-segment seems superimposable to what we defined aMdLF. These authors also found that MdLF-I and MdLF-II have their posterior termination at the SPL/PreCu and the area across the POS, respectively. We could not detect a clear anatomical separation among the more superficially located fibers of the subsegment here named pMdLF, whose fibers run from aTTG and pSTG to SPL, PreCu and across POS. Our results suggest that pMdLF fibers do originate from aTTG and pSTG and not from the anterior aspects of the temporal lobe, namely the TP. These findings are in line with the description from Wang et al. (2013) and in disagreement with the results from Kalyvas et al. (2020).

Our connectivity analysis at group level shows that aTTG is consistently connected with SPL and that PT and pSTG are connected with both SPL and sLOC. This connectivity pattern is very similar to the anatomical connections of MdLF-I and MdLF-II reported by Kalyvas et al. (2020). Therefore, we believe that the main contribution of our study to current literature is to provide a robust description of MdLF course, cortical terminations and subcomponents based on congruent data derived by high quality anatomical specimens and DTI imaging with high angular resolution.

### Functional Role of MdLF

Coming to the third controversy about MdLF, namely its functional role, no conclusive data have been provided by electrical stimulation during awake surgery (De Witt Hamer et al., 2011) nor by lesion models, such as after post-operative resection of the bundle. Therefore, MdLF involvement in language, attention, or auditory functions has been speculated on the basis of its anatomical description. In this regard, in agreement with previous anatomical studies, the most important anatomical information emerging from the present study is that the MdLF and its sub-segments seem to be the closest white matter pathways to the primary auditory cortex and the acoustic radiation.

Auditory function, whose primary (“core”) and contiguous secondary (“belts” and “parabelts”) cortical areas are located at the level of the STG, is thought to be organized into parallel streams encoding the information “what” (ventral stream), “where,” “when,” and “how” (dorsal stream) (Recanzone and Cohen, 2010; King et al., 2018; Rauschecker, 2018). These areas are surrounded by the MdLF fibers, which according to our results may support a communication of auditory information with the SPL and sLOC.

Posterior-MdLF could be related to the “where” and “what” auditory functions. Similar potential functional role have been suggested by other anatomical studies (Wang et al., 2013; Kalyvas et al., 2020). The caudal (posterior) auditory belt, located at the PT, has been shown to be functionally correlated with the LOC for decoding the direction of sound-source movements indeed, playing a central role in supporting auditory motion perception, particularly in the right hemisphere and in early blind subjects compared to sighted individuals (Lewis et al., 2011; Alink et al., 2012; Dormal et al., 2016). This cross-modal involvement of extrastriate occipital cortex with vision and hearing has been shown in both stroke lesion models and in transcranial stimulation studies (Bolognini et al., 2011, 2016). Moreover, the anatomical relationship between the acoustic radiation and the pMdLF in the temporal lobe, along with terminations of the pMdLF, AF, SLF, and IFOF within the parieto-occipital areas seems to support a possible role of pMdLF in processing of auditory information. Therefore, particularly in the dominant hemisphere, pMdLF could be involved also in the processes subtending learning of verbal stimuli. This seems also reinforced by the post-operative deficits developed by the illustrative case #2, who maintained intact learning potential for visual stimuli but showed impaired potential to learn verbal stimuli.

According to Tremblay et al. (2019), MdLF connectivity between the STG and parietal lobe suggests a role for this tract in supporting speech processing, possibly through changes in attentional biases. Other studies, owing to MdLF connectivity with parietal regions, suggest a role of the MdLF in attentional functions through a correlation between poor attention and the micro-structure of MdLF in patients with schizophrenia (Steffens et al., 2017).

The strong connection between both primary and secondary auditory areas in the STG and secondary visual areas in the occipital lobe also supports the role of the MdLF in visual-auditory integration processes (Wang et al., 2013; Kalyvas et al., 2020).

The anterior part of secondary auditory cortex, including the PP and the aSTG, is believed to process the properties of an auditory object that are not dependent upon spatial location or attention (Kaas and Hackett, 1999; Rauschecker and Tian, 2000; Tian et al., 2001; Warren and Griffiths, 2003; Arnott et al., 2004; Kusmierek and Rauschecker, 2009; Rauschecker, 2015). Higher-order temporal and occipital cortices are involved in successful encoding of cross-modal associations between common auditory and visual objects. In a fMRI study in successful encoding of semantically congruent and incongruent audio-verbal memories, the LOC was found to play a critical role in the creation of congruent memory traces, while the STG/STS contributes to the formation of incongruent memories (Naghavi et al., 2011). This network between auditory cortices and the visual ventral network may play a particularly important role in learning and in constructing memory-dependent perceptual representations of the auditory world (King et al., 2018).

Our connectivity analysis demonstrated that the LOC may be an important hub common to both MdLF branches (clearly different in cortical terminations within the temporal region). This important region has been recently suggested to be a key site for the storage of multisensory memory representations with semantically congruent elements (Murray et al., 2004, 2005; Lehmann and Murray, 2005). The LOC activation has also been showed to be involved in the formation of perceptual cross-modal associations required for the generation of congruent audio-verbal memories (Naghavi et al., 2011).

Based on our anatomical results, we can hypothesize that the predominantly perceptual associations in congruent memories and the mainly conceptual associations in incongruent memories are encoded by the higher-order occipital cortex and the lateral temporal cortex, respectively. The aMdLF may then play a crucial role within this network for retrieval of memories that are congruent and already consolidated into visual-associated categories.

### Postoperative Neuropsychological and Language Examination

The two clinical cases described in our study, provided a lesional model with new possible insights into the functional implications of the MdLF. In case # 1, the tumor was located at the aSTG and TP on the right side (Figures 9, 11a). Musical hallucinations are commonly found to originate from temporal regions in both the primary and secondary auditory cortices of either of hemispheres in patients with schizophrenic disorders or brain lesions (Coebergh, 1990; Lennox et al., 2000; Braun et al., 2003; Bernardini et al., 2017). In our patient, the original type of hallucinations (melodies) together with the anatomical location and the possible white matter connectivity underlying the symptoms confirm the role of non-dominant hemisphere in this type of phenomena (Critchley, 1977; Clynes, 1982; Berrios, 1990; Lennox et al., 2000; Braun et al., 2003; Stewart and Brennan, 2005). However, after surgical lesion of aMdLF fibers, we could not document an impairment of visual-auditory integration nor of audio-verbal memories, which are some of aMdLF presumed functions.

**FIGURE 12:**
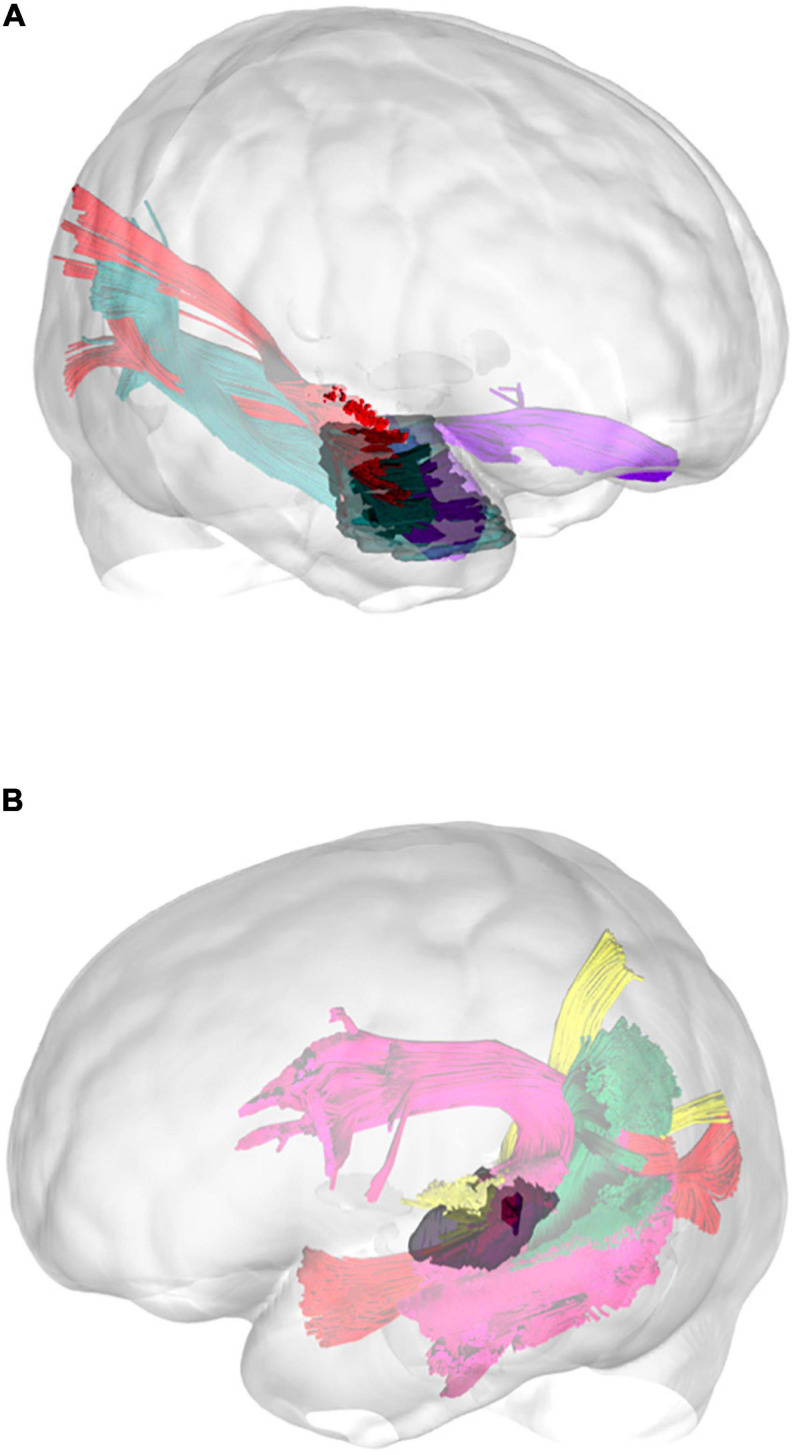
Three-dimensional reconstruction of the connectivity possibly affected by the tumors in illustrative case #1 **(A)** and case #2 **(B)**. In the first patient, the tumor directly affected the connectivity of the UF, ILF, and aMdLF on the right side. In the second case, the reconstructed tumor volume displayed a possible infiltration of the pMdLF, short fibers of the AF, and the vSLF.

The most important postoperative change regarded the semiology of the hallucinations, namely the onset of musical obsessions, an uncommon but previously described finding (Zungu-Dirwayi et al., 1999; Stewart and Brennan, 2005; Bleich-Cohen et al., 2011; Orjuela Rojas and Lizarazo Rodríguez, 2018). The relevant scientific literature suggests that in psychotic auditory hallucination, the most likely mechanism is the release of inhibition of the auditory cortex by other cortical auditory neural assemblies (Hugdahl, 2009, 2017), including those in the contralateral hemisphere (Ait Bentaleb et al., 2002; Braun et al., 2003). Merging our anatomical results within the connectomic analysis, we may interpret the patient’s symptoms as a loss of balance between the anterior portion of the auditory network within the anterior temporal lobe (UF-anterior, ILF, and aMdLF, Figure 12A) and the posterior portion (acoustic radiation, pMdLF, AF, SLF, IFOF, ILF) on the right hemisphere (Hugdahl, 2009, 2017). This altered communication probably resulted in a prominent activation of right aTTG, with change in quality of hallucination sounds, and in reduced inhibition of auditory areas by frontal areas due to lesion of UF and extreme capsule fibers, causing an obsessive behavior (Berrios, 1990; Zungu-Dirwayi et al., 1999; Hugdahl, 2009, 2017; Lagemann et al., 2012; Orjuela Rojas and Lizarazo Rodríguez, 2018).

Despite the interesting association between MdLF and auditory hallucinations, it is mandatory to interpret these results with caution because the postoperative clinical situation in our patient may be also related to other psychological/psychiatric causes not related to postsurgical structural modifications.

Case #2 displayed a low-grade astrocytoma that involved the pSTG, PT, and aTTG on the left side. Also this patient experienced auditory hallucinations (*“intermittent whistling sound inside my head”*), confirming the common origin for this phenomenon within the STG (Coebergh, 1990; Lennox et al., 2000; Braun et al., 2003; Bernardini et al., 2017). The main white matter pathways involved in the tumor area were the pMdLF and the short fibers of the AF (Figures 10, 11b, 12B). Merging this anatomical substrate with the results from speech and neuropsychological tests (impaired learning, memory, and concentration), we may reinforce our hypothesis regarding the functional network supported by the pMdLF. Acoustic information would be processed within the aTTG and subsequently redirected by the pMdLF to SPL and LOC, while the AF would support the phonological processing of verbal information, word flow, and verbal working memory through its terminations within the temporo-parietal and frontal regions (Wang et al., 2013; Fernández-Miranda et al., 2015; Bernard et al., 2019; Tremblay et al., 2019). The language impairment identified in this patient confirms the functional evidence regarding the AF and its close anatomical relationship with the primary auditory cortex.

However, since the patient demonstrated how the phonological repetition was used as a compensatory mechanism to overcome verbal learning impairment, we may suspect that the phonological/repetition loop (supported by the AF) was still intact at the neuropsychological examination. This case demonstrates how the AF and the MdLF pathways may work, processing in parallel auditory information with differences in functional domains. We believe that the pMdLF, connecting primary auditory areas and parieto-occipital regions, may play a key role within auditory “what and where” integration processes, which are necessary for consolidation of cross-modal inputs during verbal learning.

This case shows a different anatomical substrate for verbal and visual learning abilities. The selective impairment of auditory processing, with an intact visual memory and learning both pre- and post-operatively, indicates that the pMdLF is a specialized pathway within the auditory network.

We believe that verbal learning impairment was then correlated with a damage of dorsal auditory network including primary afferent fibers to aTTG, the pMdLF and its connections with the SLF, IFOF, and AF within the temporo-parieto-occipital area on the left side.

### Limitations

Our study has some limitations. The first one concerns the anatomical comparison between diffusion tensor tractography (DTT) in young healthy volunteers and white matter dissection of post-mortem brains. The different age spans of the two populations represent a limitation when interpreting the results. Hence, a quantitative comparison between these two techniques may be difficult to achieve and was beyond the aim of this paper. Our aim was to determine, in post-mortem brains, the organization of MdLF fibers and provide evidence for a better orientation within the temporo-parieto-occipital connections. In addition, despite the classical limitations of white matter dissection and the high risk of artifacts during the dissection of regions in which the fibers intermingle or overlap (parietal and occipital lobe cortical terminations), the information provided by the dissection is consistent with other studies using the same technique and high-quality specimens (Maldonado et al., 2013; Wang et al., 2013; Latini et al., 2017).

Further, using normalized anatomical ROIs and results in a group analysis may create false results. In our opinion, the use of atlas-based regions to create connectivity maps minimizes the risk for incongruent/inconsistent anatomical results. We decided to use normalized percentage of streamlines within the regions and display them with a cut-off of 5% to remove possible artifacts. However, the risk of incomplete reconstruction or the effect of kissing/crossing fibers in complex anatomical region such as temporo-parietal-occipital region or the SS cannot be excluded with the current techniques. This may create tractography “contaminations,” with resulting overestimation of volume and difficulties to determine cortical terminations. Indeed, despite we performed a meticulous microdissection and used a strict, advanced tractographic method, we cannot exclude that the absence of MdLF terminations at the IPL represents a false negative from both techniques. On the other hand, the intrinsic advantage of high angular diffusion tensor imaging (DTI) and proved optimal quality of cadaver specimens may be reduce the risk of false or incomplete results. Nonetheless, our findings seem to strengthen previous findings that report a lack of significant MdLF connections with the IPL (SMG and AG).

Finally, the use of two clinical cases to support anatomical-functional results can raise criticism because of the inter-individual variability in patients with low-grade gliomas. Even though the lesions (normalized within MNI space) may result in minimal anatomical differences compared to patient-specific space, we believe that these two cases support complement anatomical information regarding the MdLF anatomy with functional/clinical implications (such as the type and origin of auditory hallucinations) from language/neuropsychological examinations. Aware of the low level of evidence provided by only two neurosurgical cases without direct cortical subcortical mapping, we invite the reader to carefully interpret our inferences regarding the functional implications of MdLF. On the other hand, we believe that the results from a very extensive preoperative and postoperative assessment support the role of MdLF in auditory networks in the presented cases. Since the scientific community is still lacking a validated intraoperative test to demonstrate the on-line inhibition of MdLF, we believe that our result may encourage further studies to develop one or more dedicated tests for intraoperative mapping of auditory functions. Further studies with larger populations of selected cases and tailored intraoperative functional tasks would be able to confirm our theories on the functional role of MdLF.

## Conclusion

Our results from post-mortem white matter dissection, subject-by-subject *in vivo* DTT, and clinical cases revealed a clear, constant, and detailed organization of the MdLF fibers in the temporo-parieto-occipital region. The main body of the MdLF is formed by an anterior-ventral segment (aMdLF) and a posterior-dorsal segment (pMdLF). The anatomical connectivity pattern and quantitative differences between the MdLF subcomponents support a pivotal role of the MdLF in supporting high order functions related to acoustic information. Due to the anatomical position and connectivity, the posterior portion, especially on left side, is possibly involved in learning process for verbal-auditory cross-modal integration. The anterior portion is on the other hand possibly involved in processing/retrieving auditory information already consolidated.

## Data Availability Statement

The datasets for this article are not publicly available due to privacy and ethical restrictions. Requests to access the datasets should be directed to the corresponding author.

## Ethics Statement

The studies involving human participants were reviewed and approved by Regional Ethical Vetting Board in Uppsala, Sweden (Dnr 2014/468). The patients/participants provided their written informed consent to participate in this study. Written informed consent was obtained from the individual(s) for the publication of any potentially identifiable images or data included in this article.

## Author Contributions

FL, GT, MF, and MR contributed to the experimental design and its implementation. FL, GT, and MF contributed to the analysis and interpretation of the data. All authors contributed to writing the manuscript at draft and any revision stages, read and approved the final version.

## Conflict of Interest

The authors declare that the research was conducted in the absence of any commercial or financial relationships that could be construed as a potential conflict of interest.

## Footnotes

1 http://dsi-studio.labsolver.org

2 http://dsi-studio.labsolver.org/download-images

3 http://mkweb.bcgsc.ca/tableviewer/visualize/

## Supplementary Material

Supplementary Material 1 Tractographic results of the HCP subjects 1–12. The two subsegments of MdLF are displayed in red (anterior segment, aMdLF) and in yellow (posterior segment, pMdLF) on sagittal and axial T1-MR images. L: left side; R: right side.

Supplementary Material 2 Tractographic results of the HCP subjects 12–24. The two subsegments of MdLF are displayed in red (anterior segment, aMdLF) and in yellow (posterior segment, pMdLF) on sagittal and axial T1-MR images. L: left side; R: right side.

Supplementary Material 3 Summary of the neuropsychological and language tests administered pre- and postoperatively in patients with low-grade gliomas.

## Abbreviations

AF: arcuate fasciculus
sLOC, lateral occipital cortex: superior part
iLOC, lateral occipital cortex: inferior part
DTI: diffusion tensor imaging
DTT: diffusion tensor tractography
FA: fractional anisotropy
ILF: inferior longitudinal fasciculus
IFOF: inferior fronto-occipital fasciculus
MdLF: middle longitudinal fasciculus
MOG: middle occipital gyrus
MRI: magnetic resonance imaging
OR: optic radiation
ROA: region of avoidance
ROI: region of interest
vSLF, superior longitudinal fasciculus: vertical component
hSLF, superior longitudinal fasciculus: horizontal component
PP: planum polare
POS: parieto-occipital sulcus
PT: planum temporale
TP: temporal pole
aTTG: anterior transverse temporal gyrus
SPL: superior parietal lobule
SS: sagittal stratum
aSTG, superior temporal gyrus: anterior portion
pSTG, superior temporal gyrus: posterior portion
UF: uncinate fasciculus
VO: vertical occipital fasciculus.
