# Supplementary figures and images for: New Insights Into the Anatomy, Connectivity and Clinical Implications of the Middle Longitudinal Fasciculus
Source: Front Neuroanat. 2021 Jan 29;14:610324. doi: 10.3389/fnana.2020.610324 (PMC7878690; doi:10.3389/fnana.2020.610324)

HCP-Subjects

aMdLF

pMdLF

L

R

L

R

HCP-#1

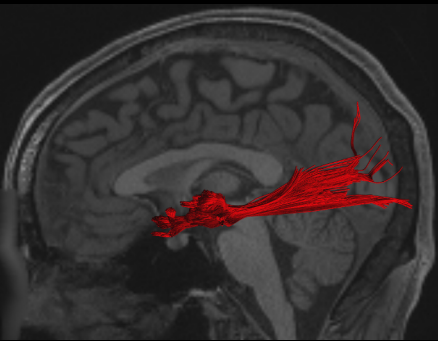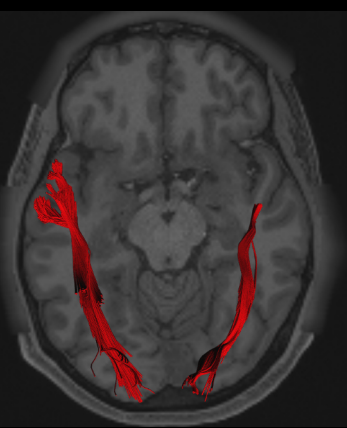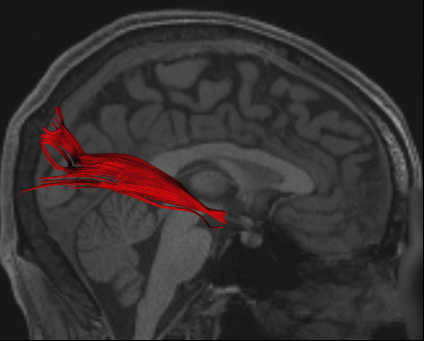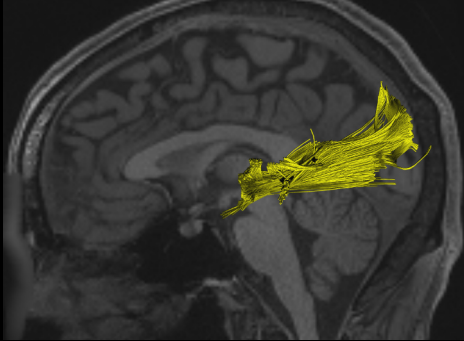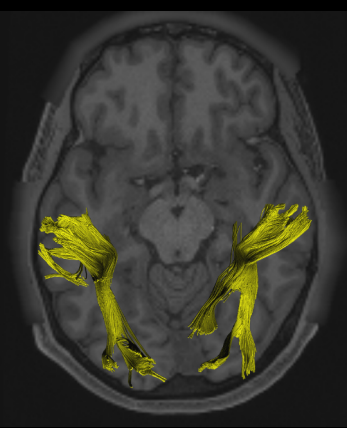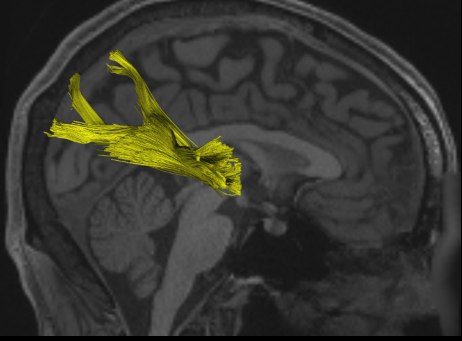

HCP-#2

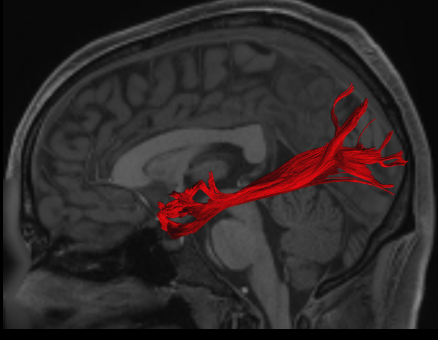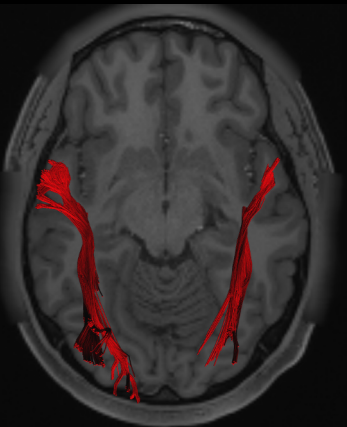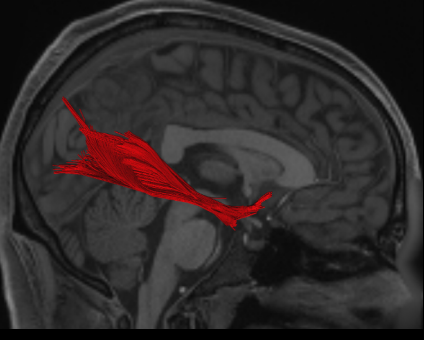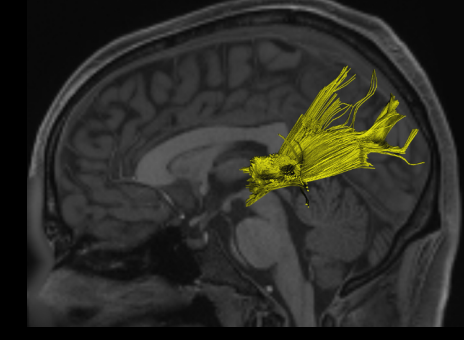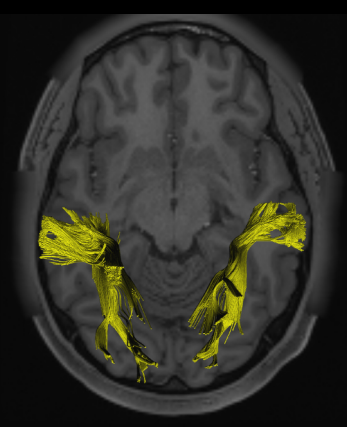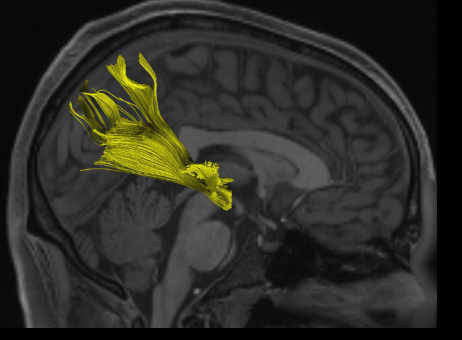

HCP-#3

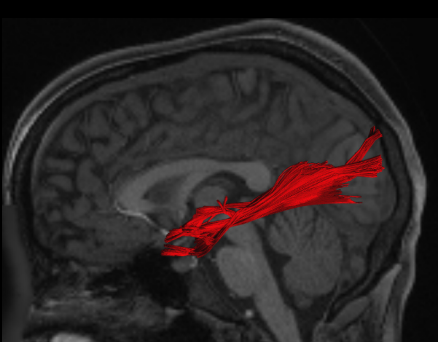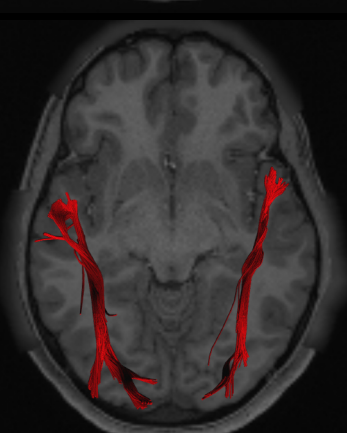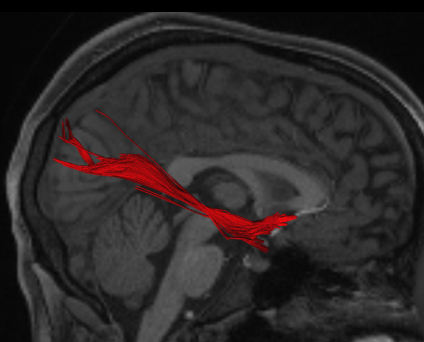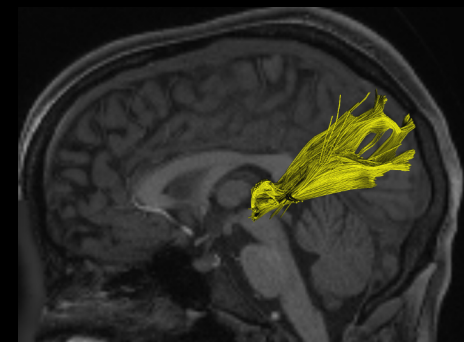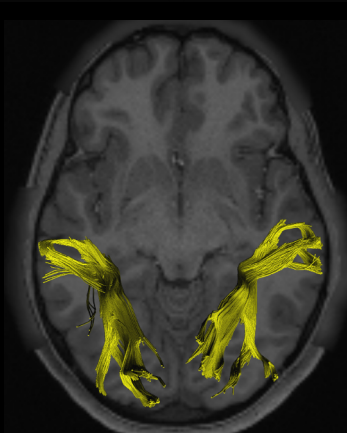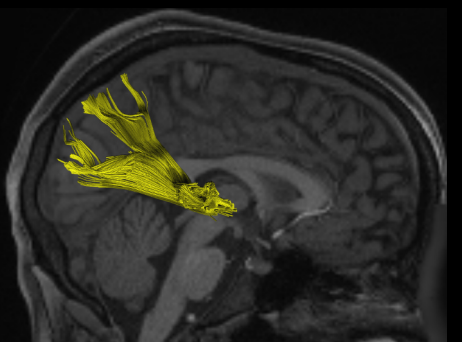

HCP-#4

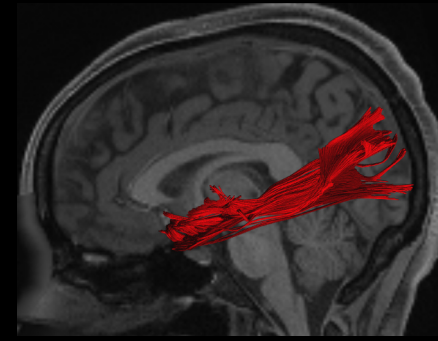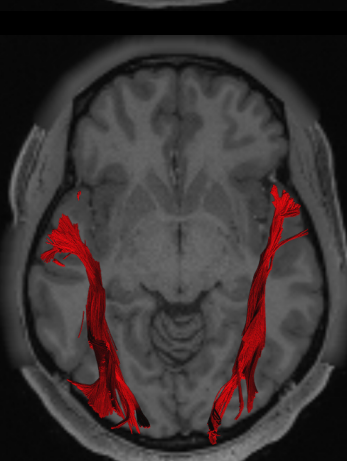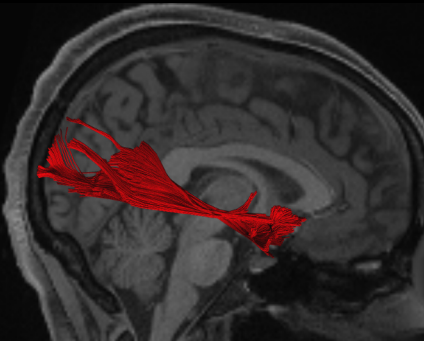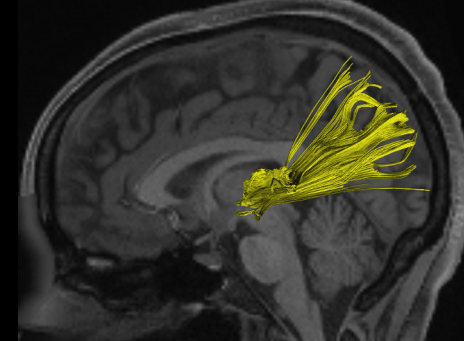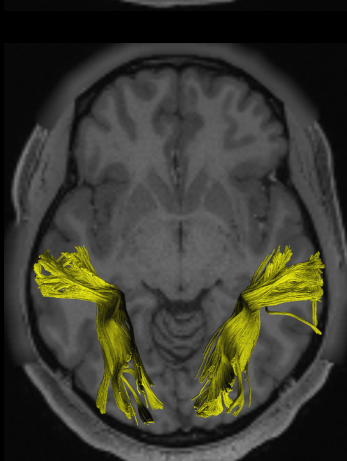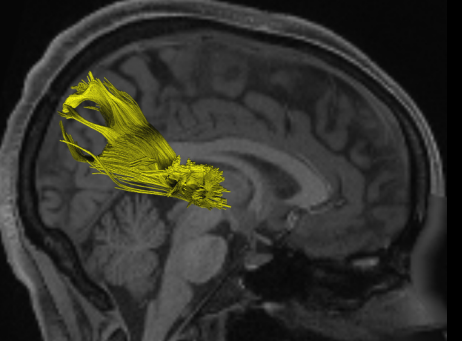

HCP-#5

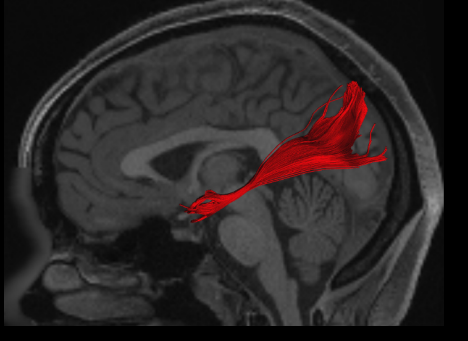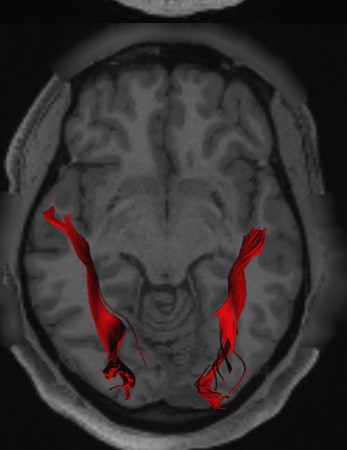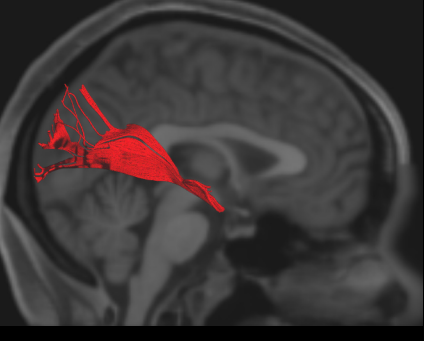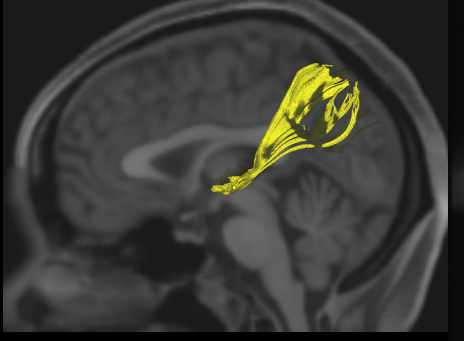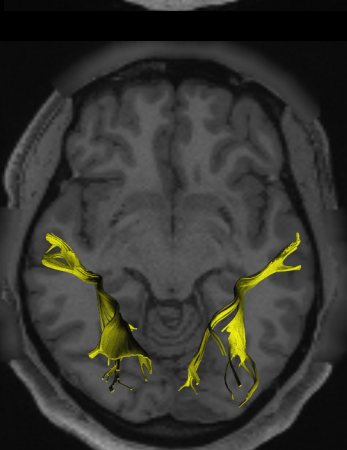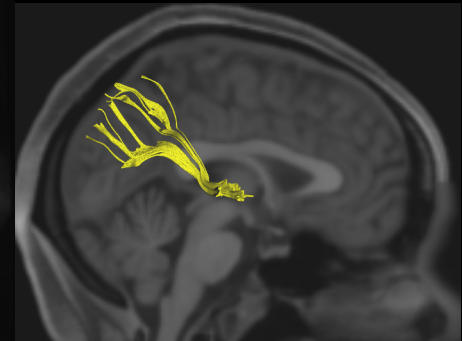

HCP-#6

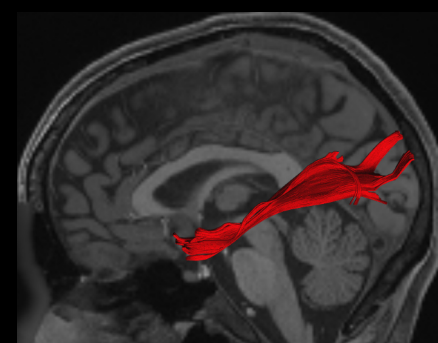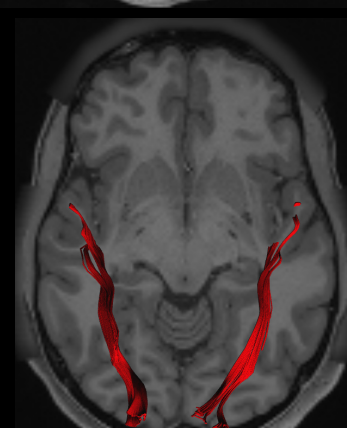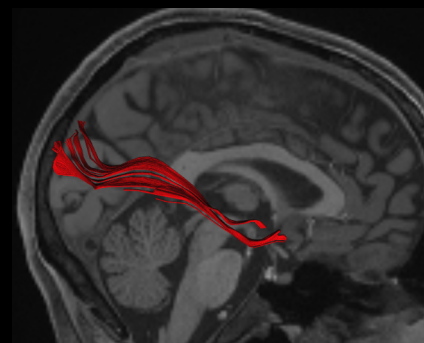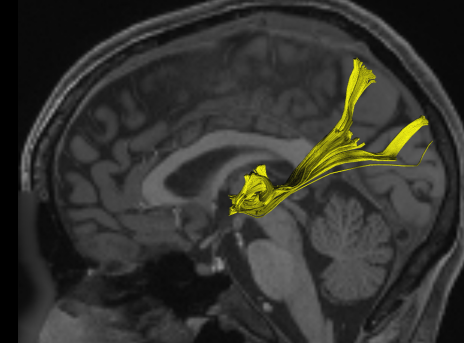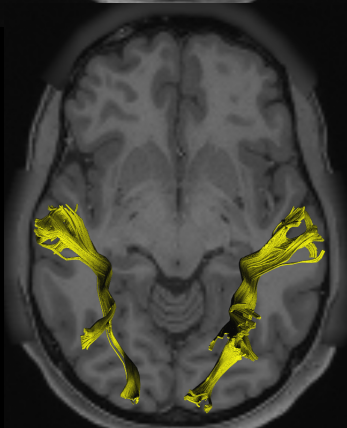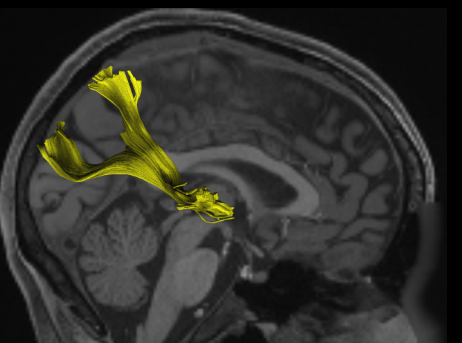

HCP-#7

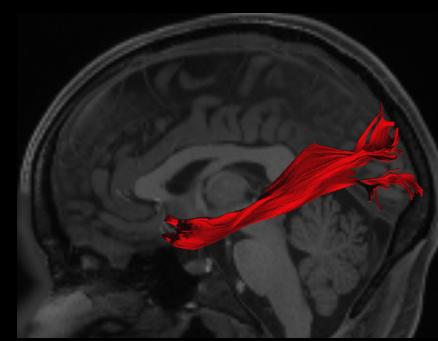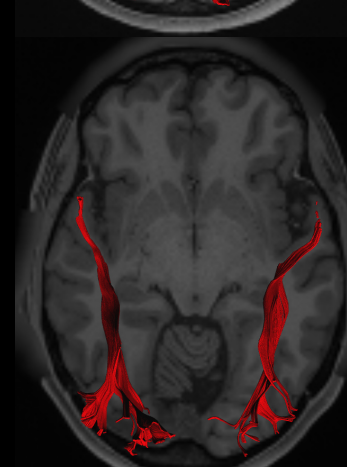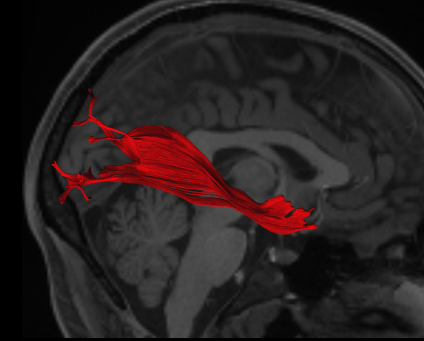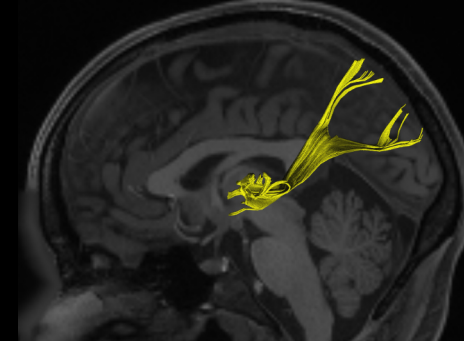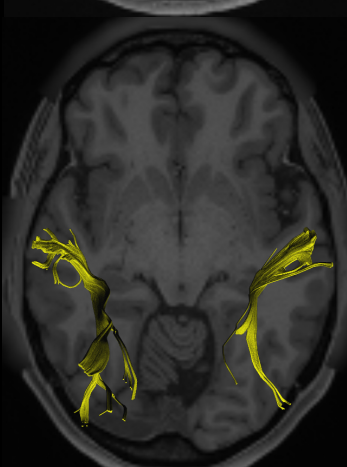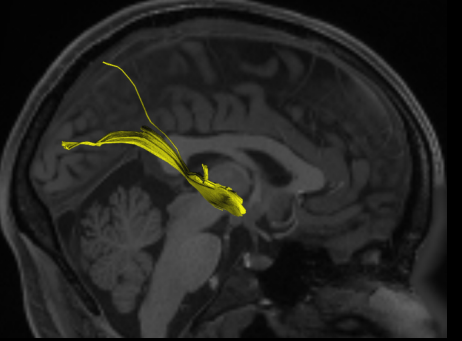

HCP-#8

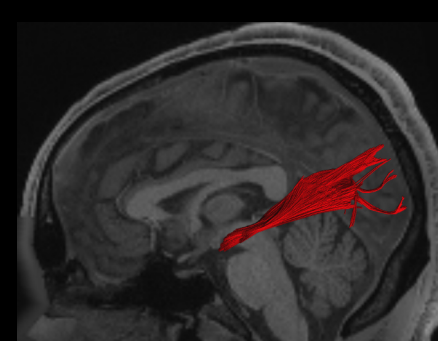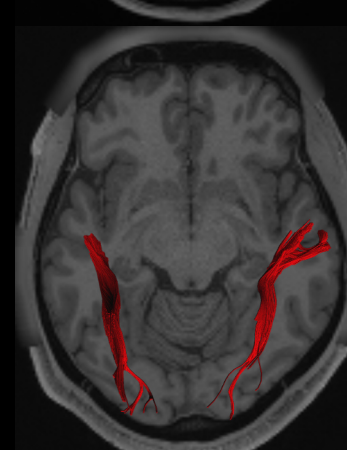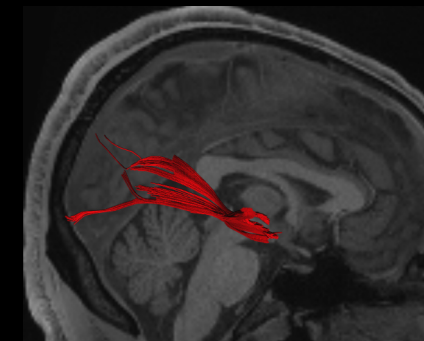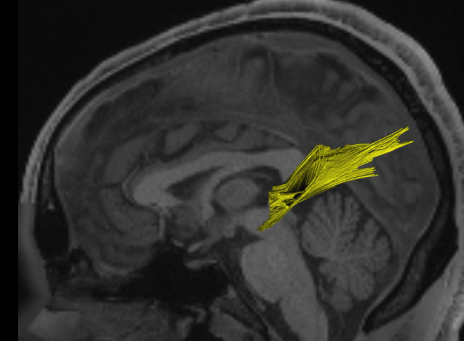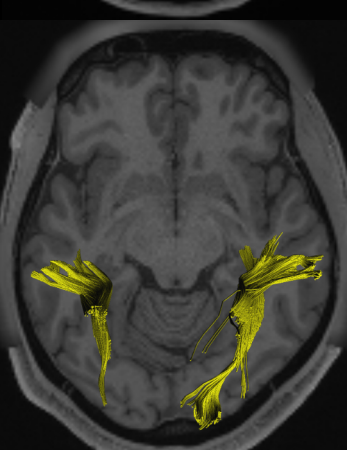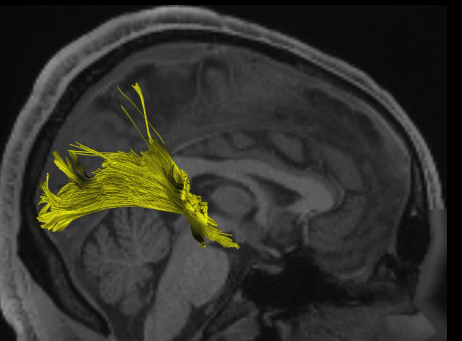

HCP-#9

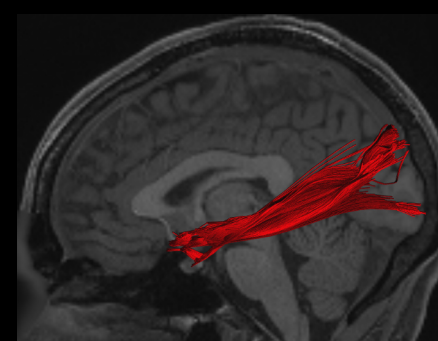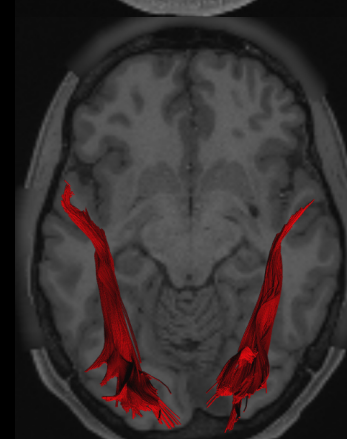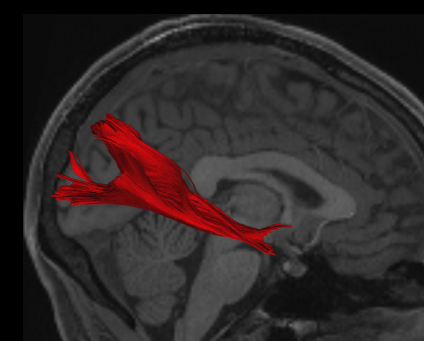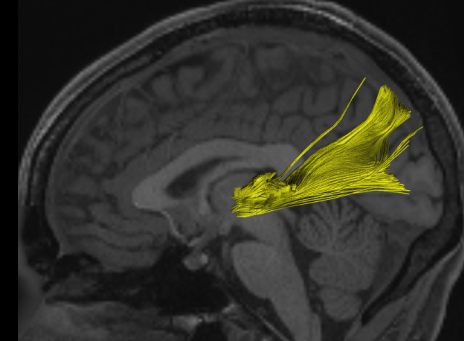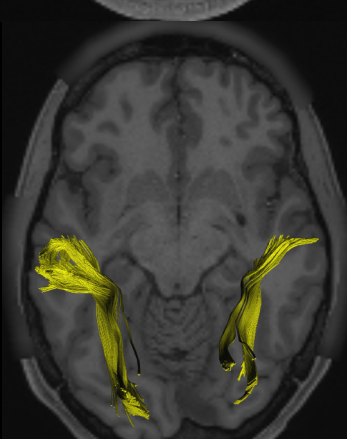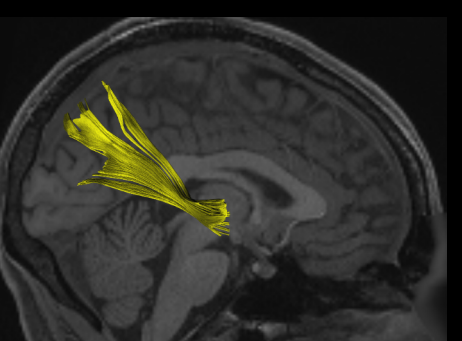

HCP-#10

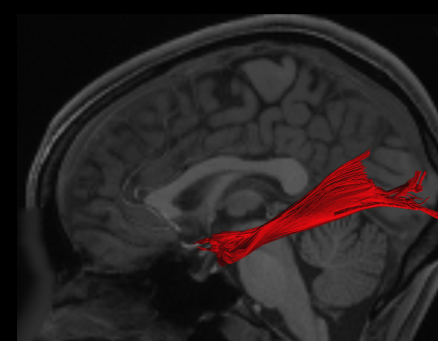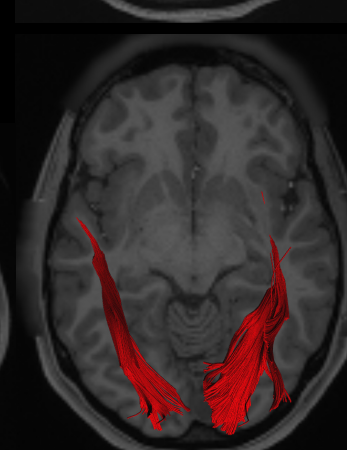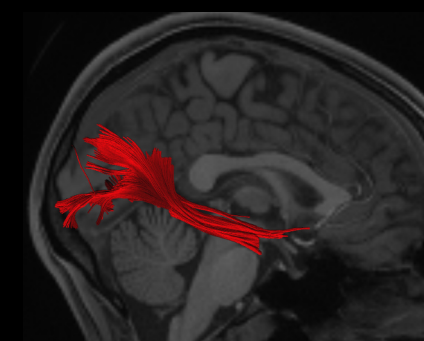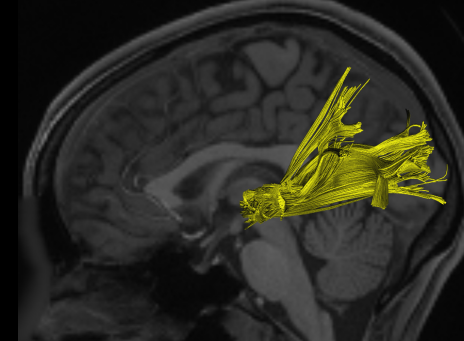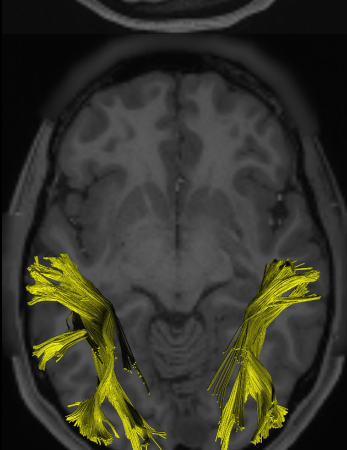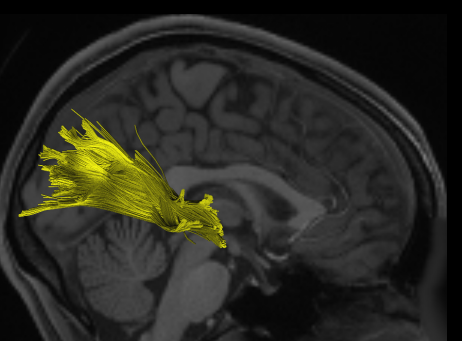

HCP-#11

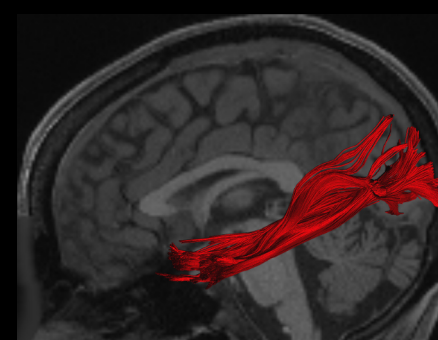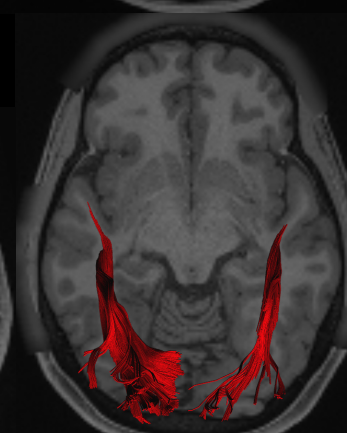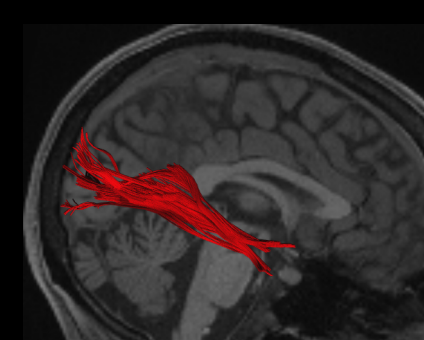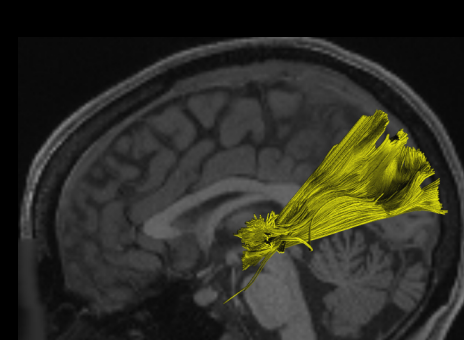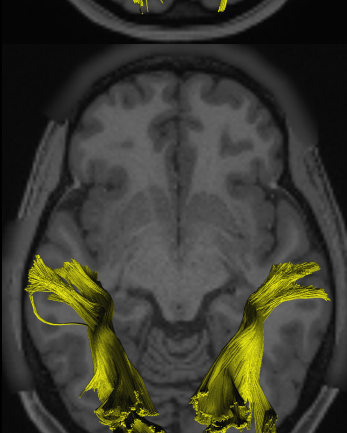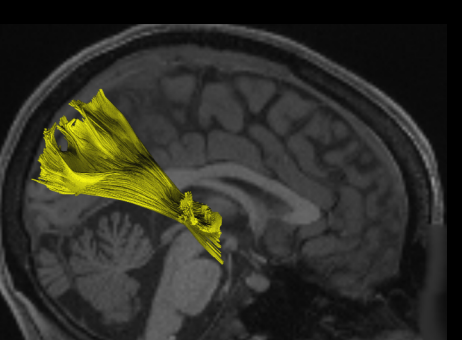

HCP-#12

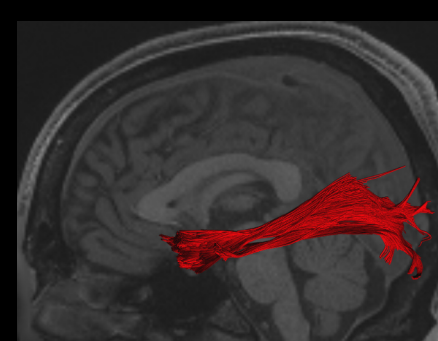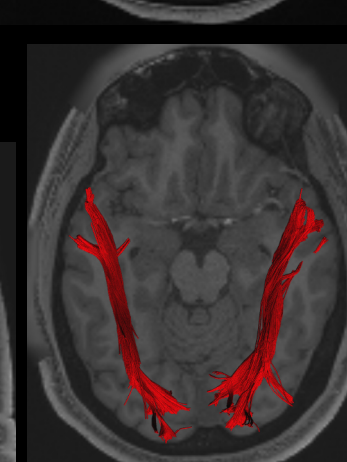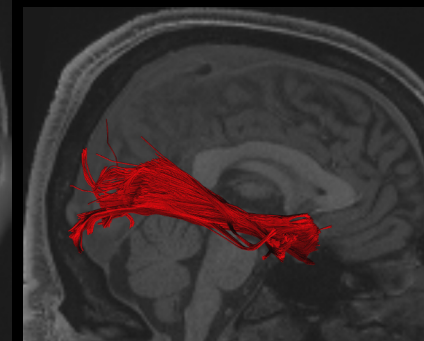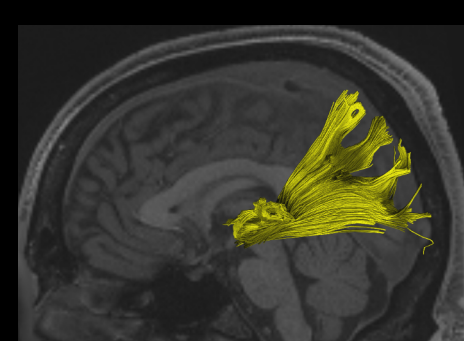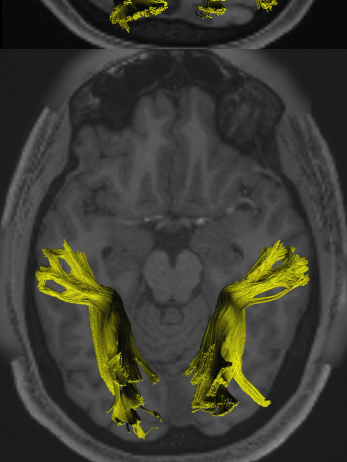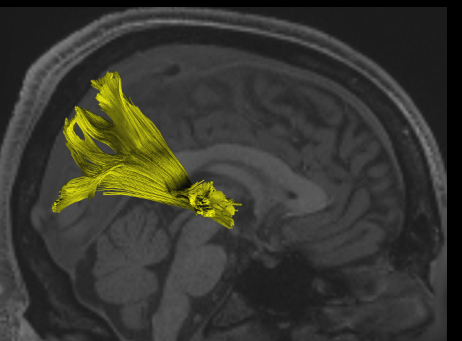

Supplement: Supplementary Material 1 — Tractographic results of the HCP subjects 1–12. The two subsegments of MdLF are displayed in red (anterior segment, aMdLF) and in yellow (posterior segment, pMdLF) on sagittal and axial T1-MR images. L: left side; R: right side. [file Data_Sheet_1.PDF]

HCP-Subjects

aMdlF

pMdlF

L

R

L

R

HCP-#13

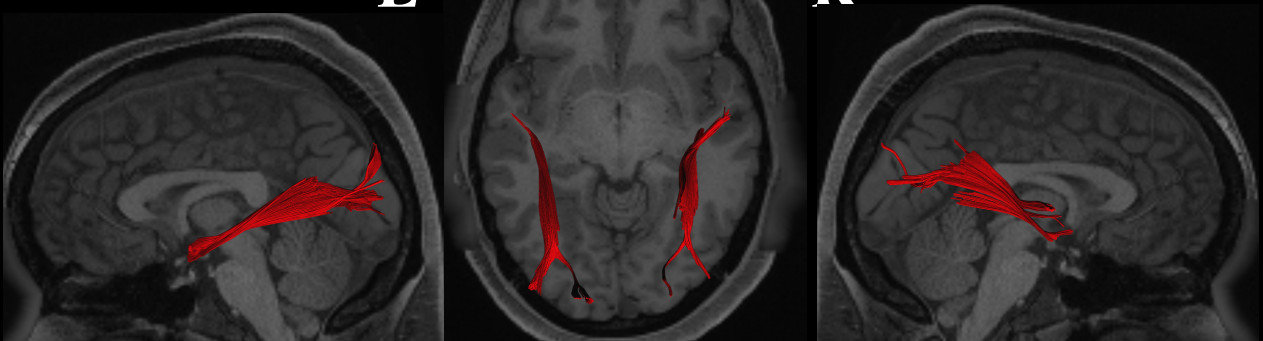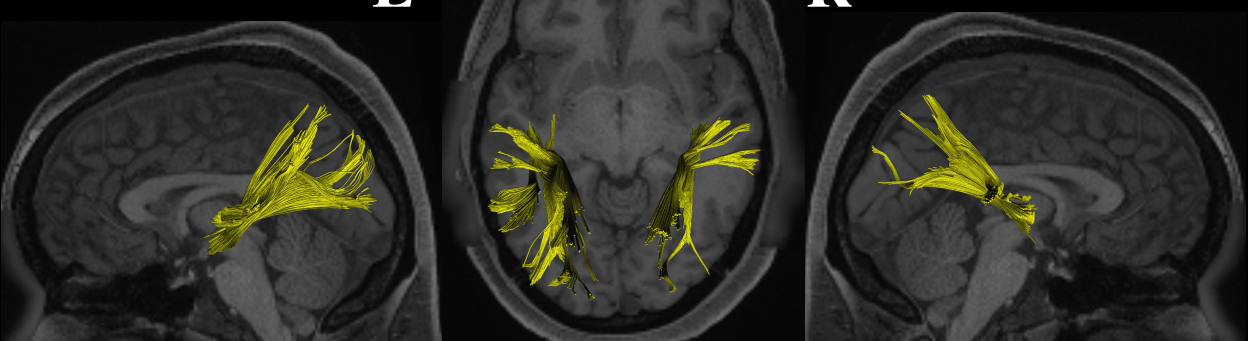

HCP-#14

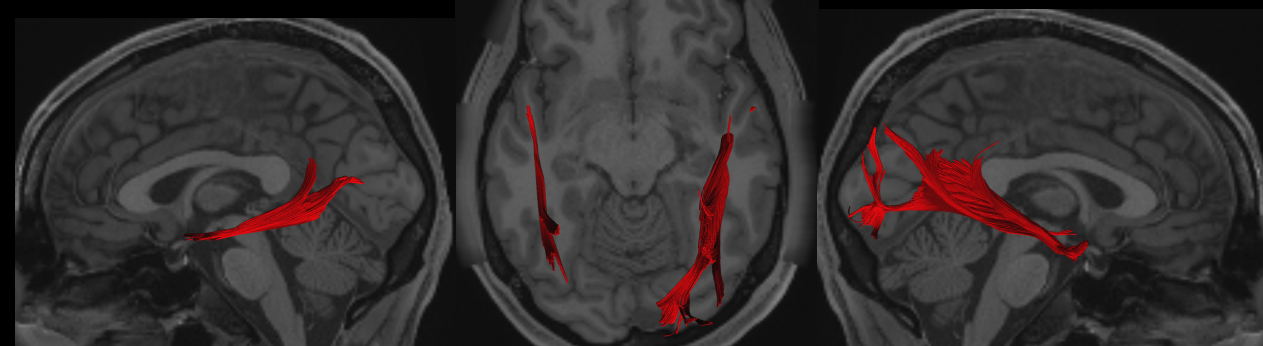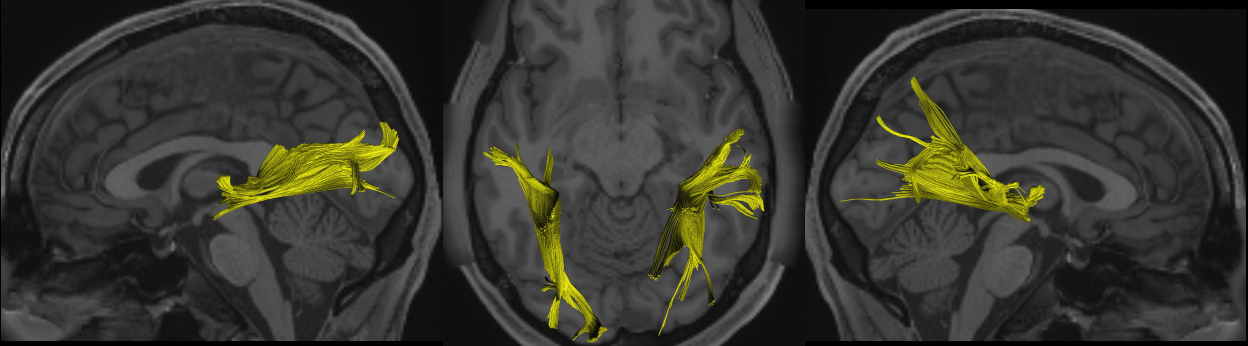

HCP-#15

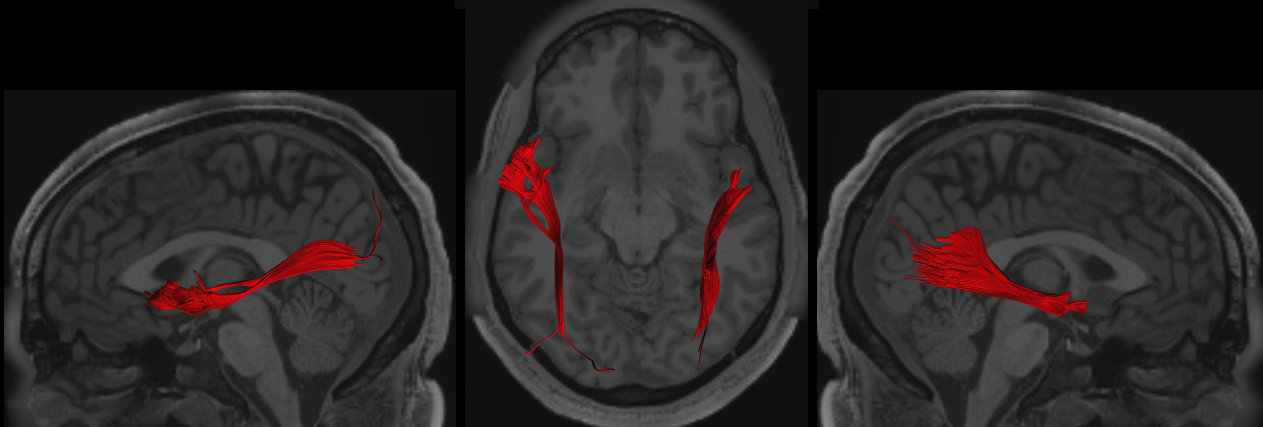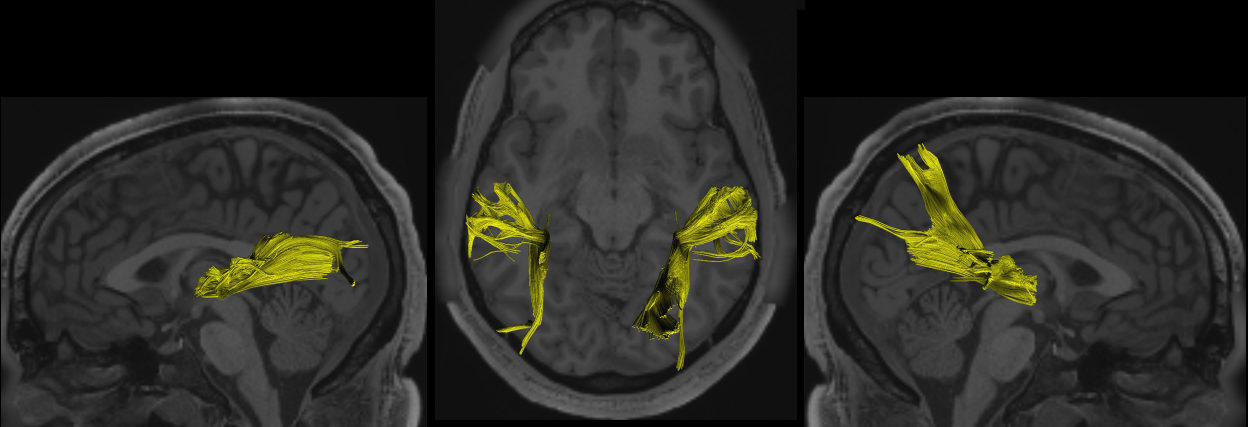

HCP-#16

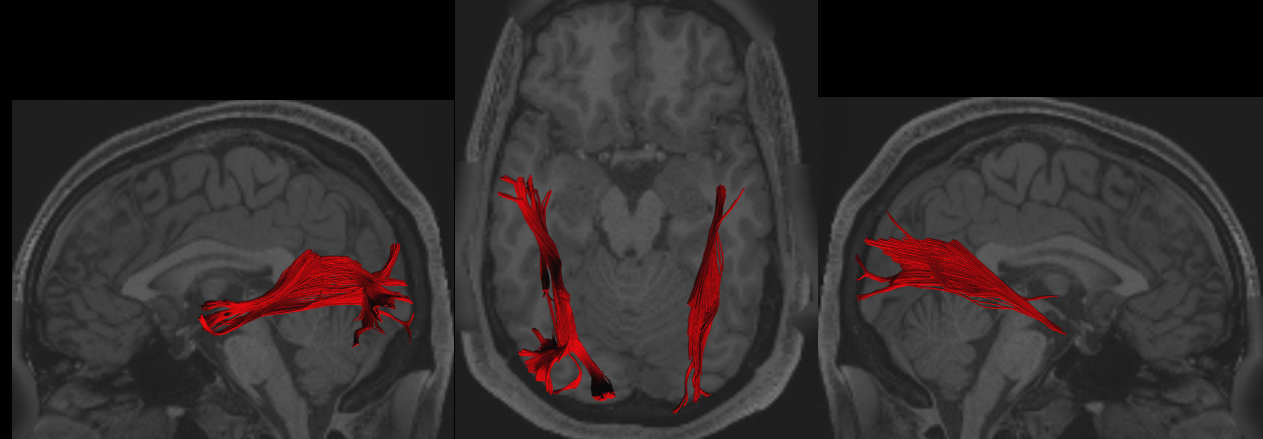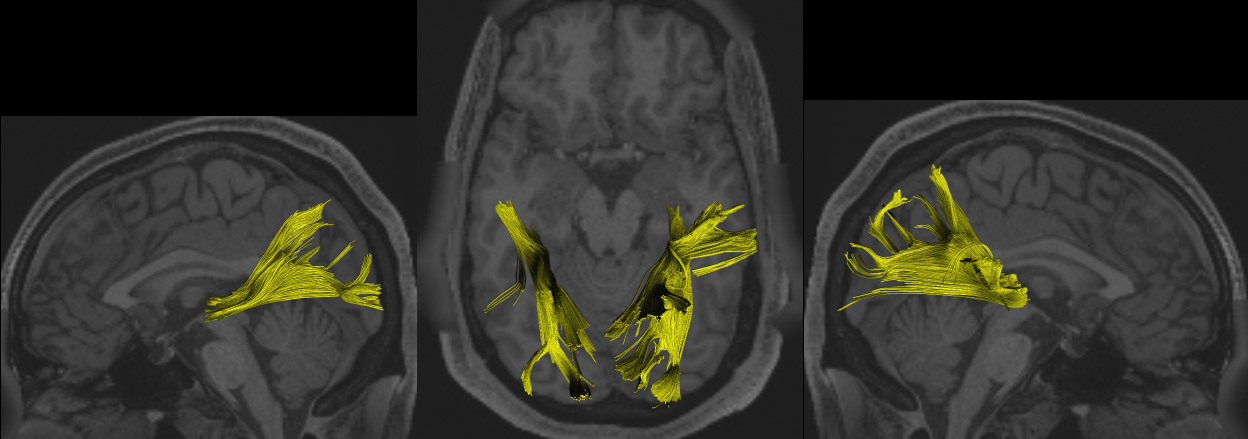

HCP-#17

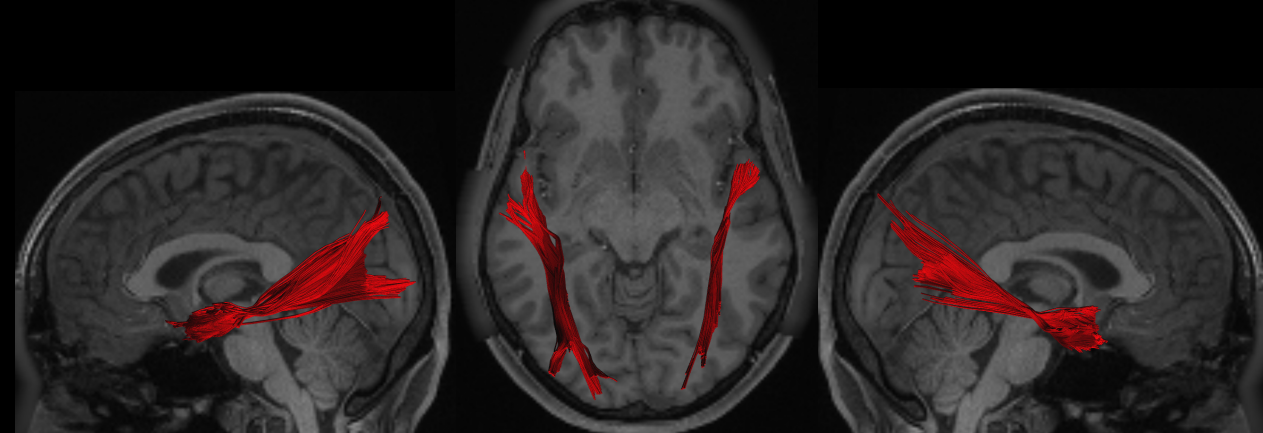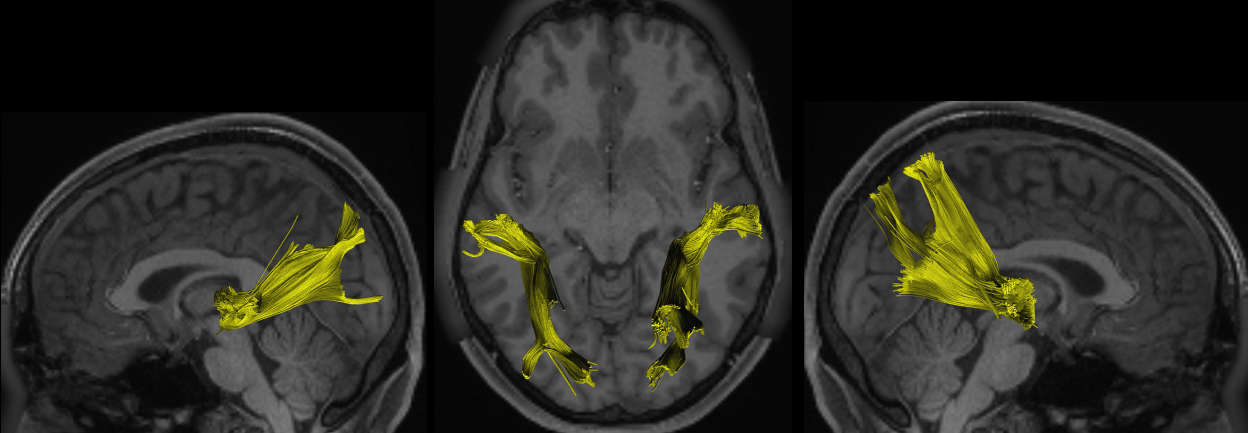

HCP-#18

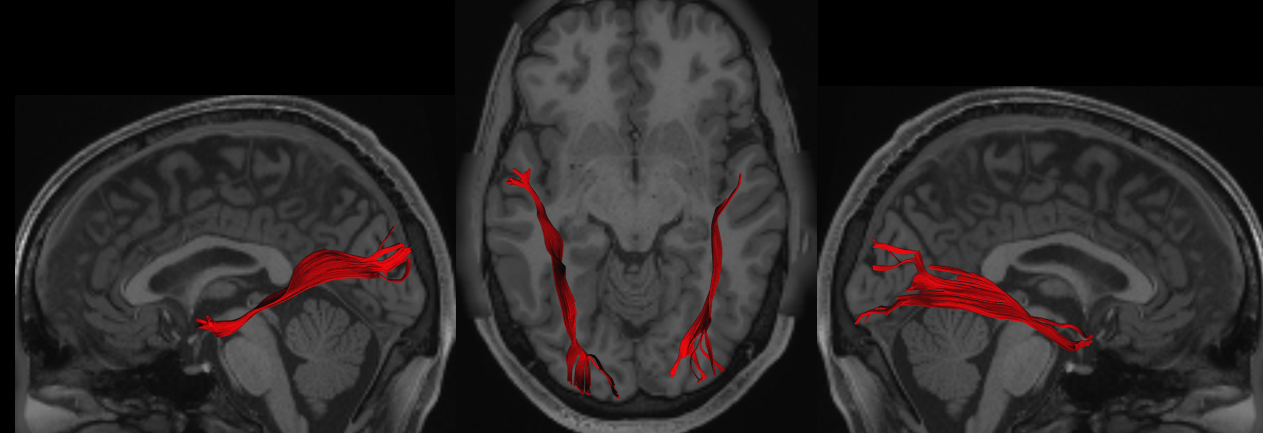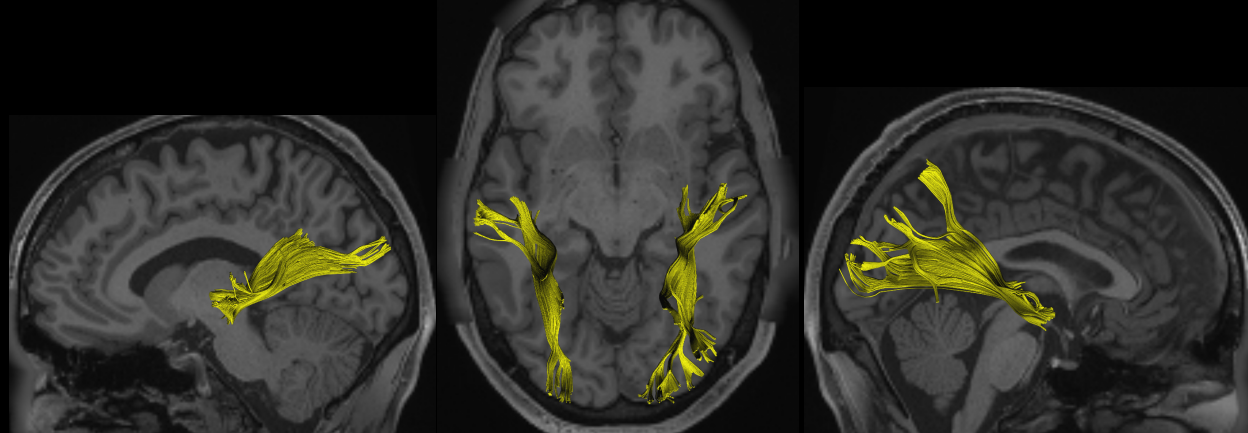

HCP-#19

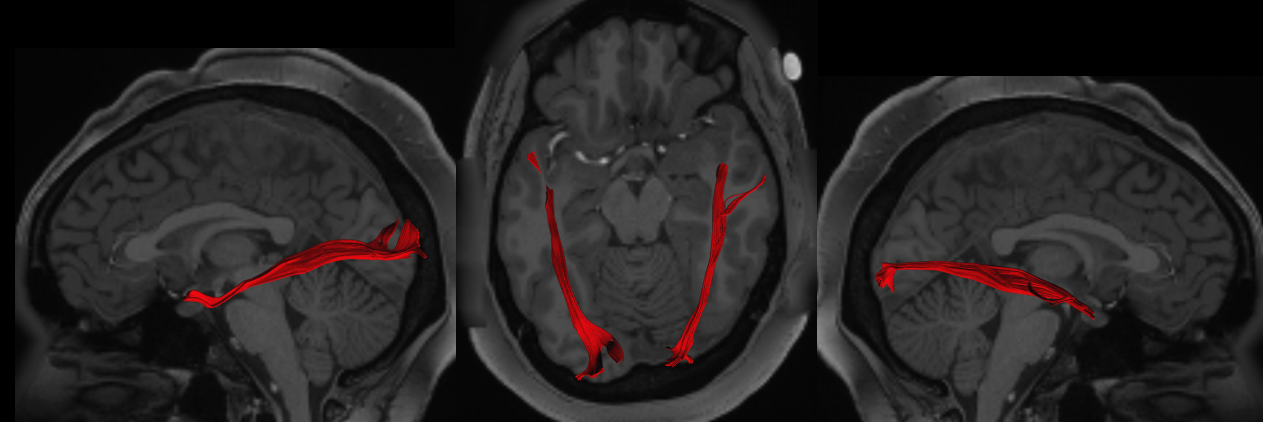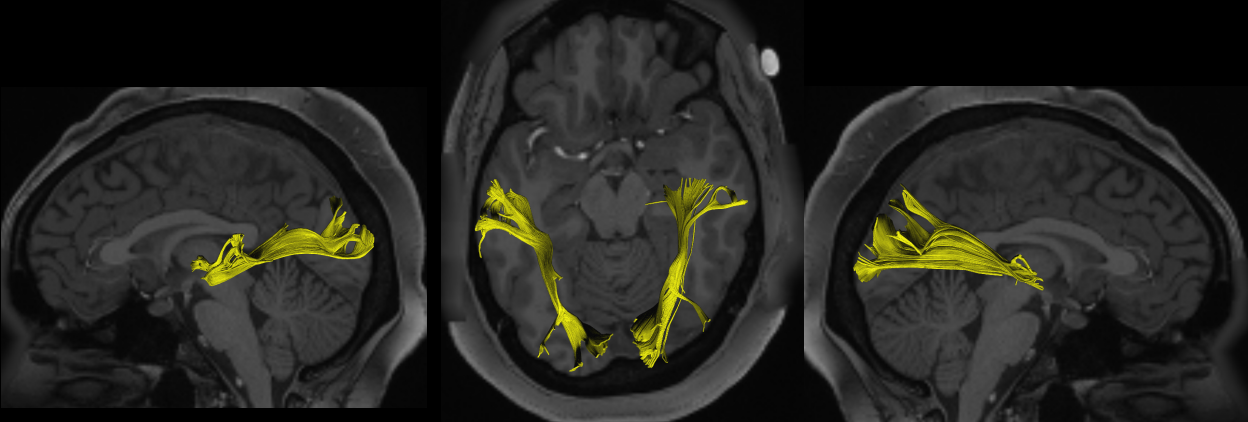

HCP-#20

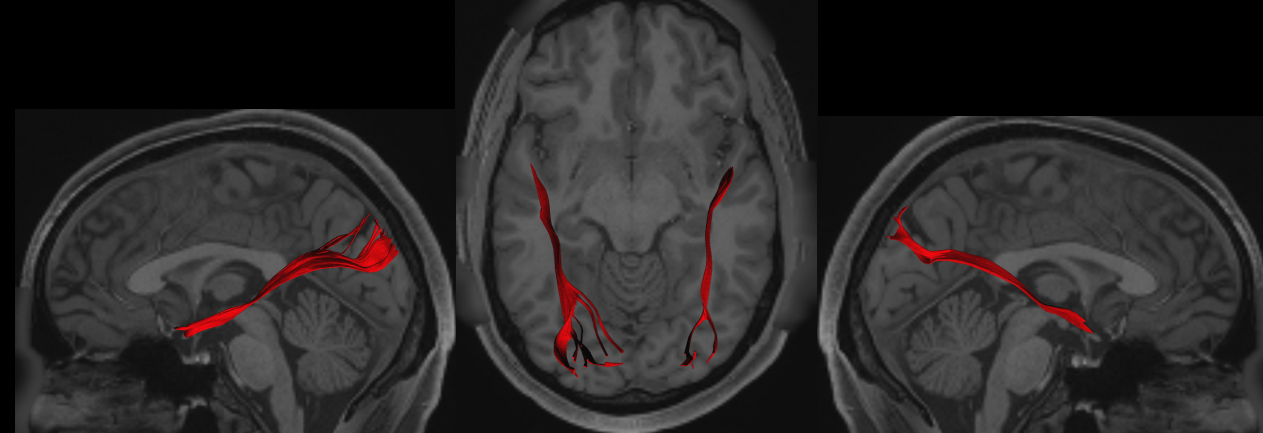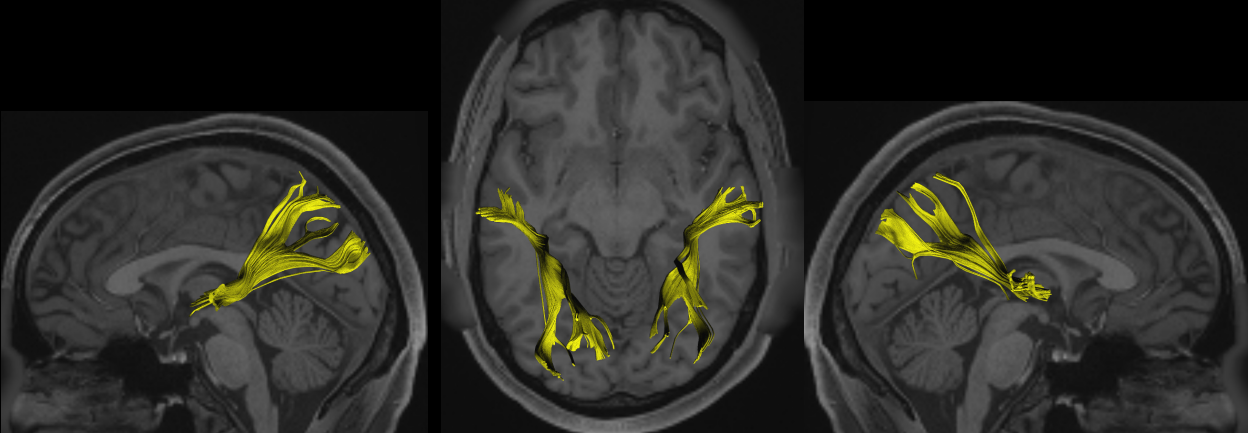

HCP-#21

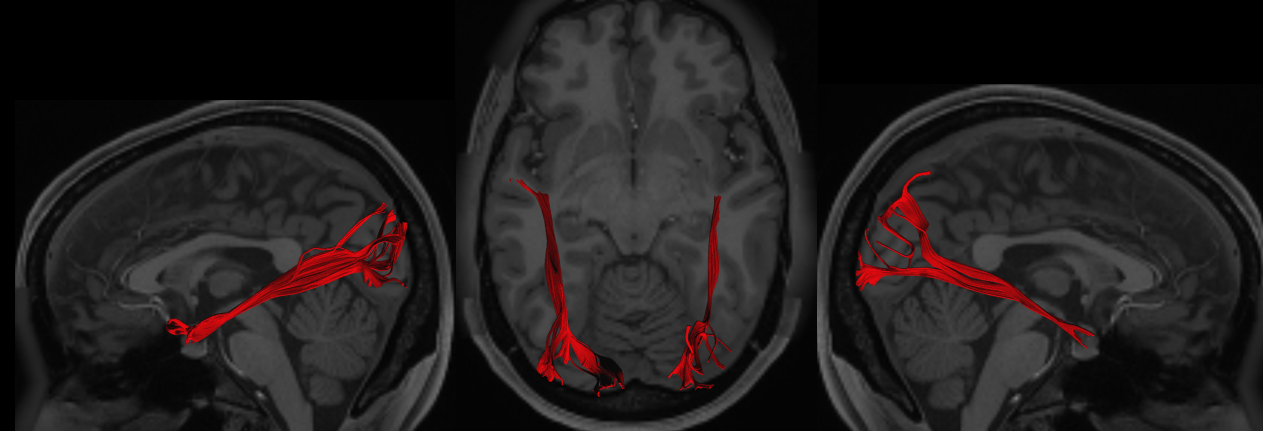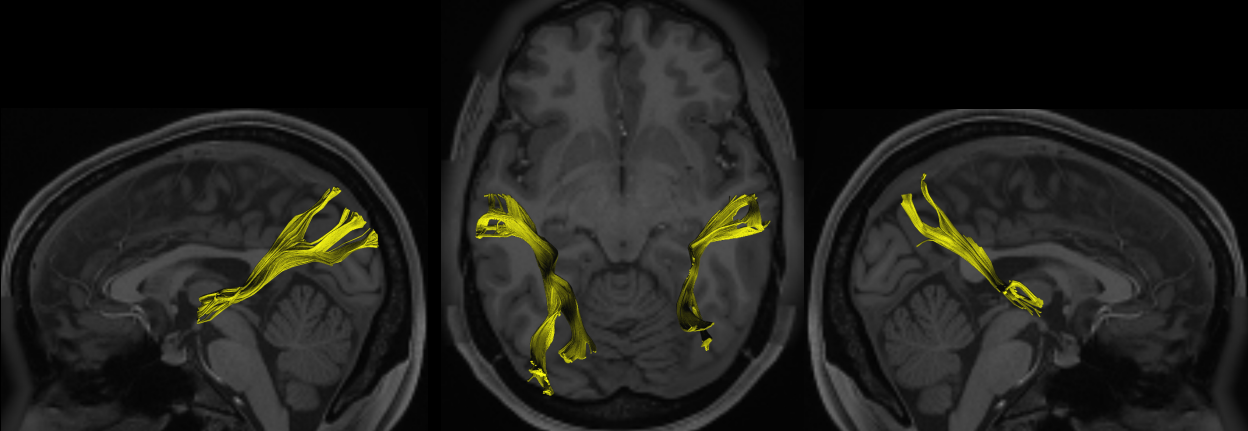

HCP-#22

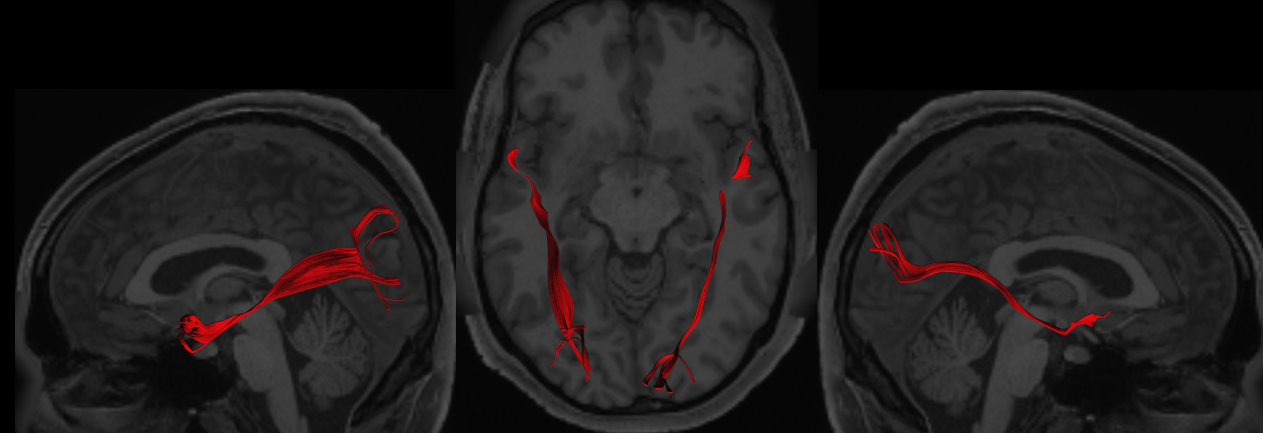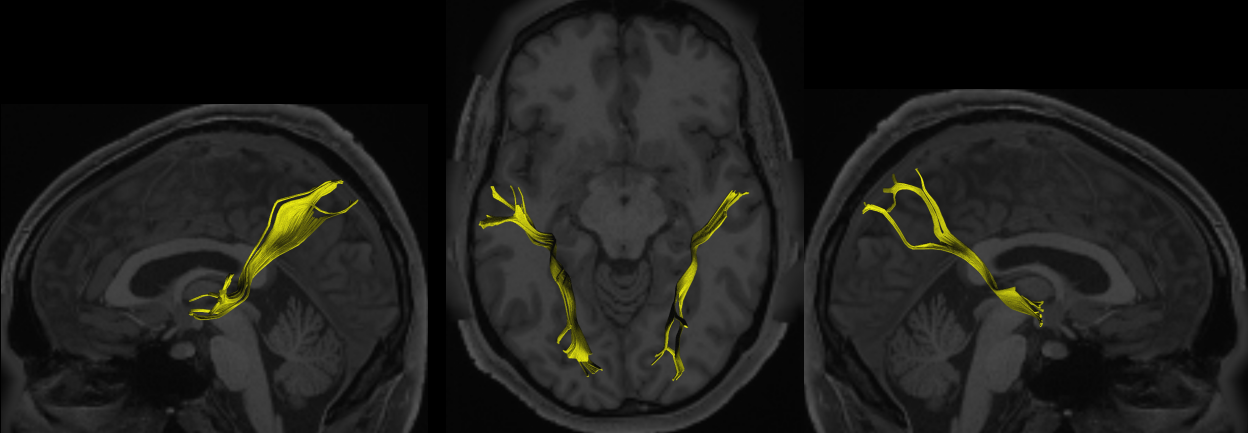

HCP-#23

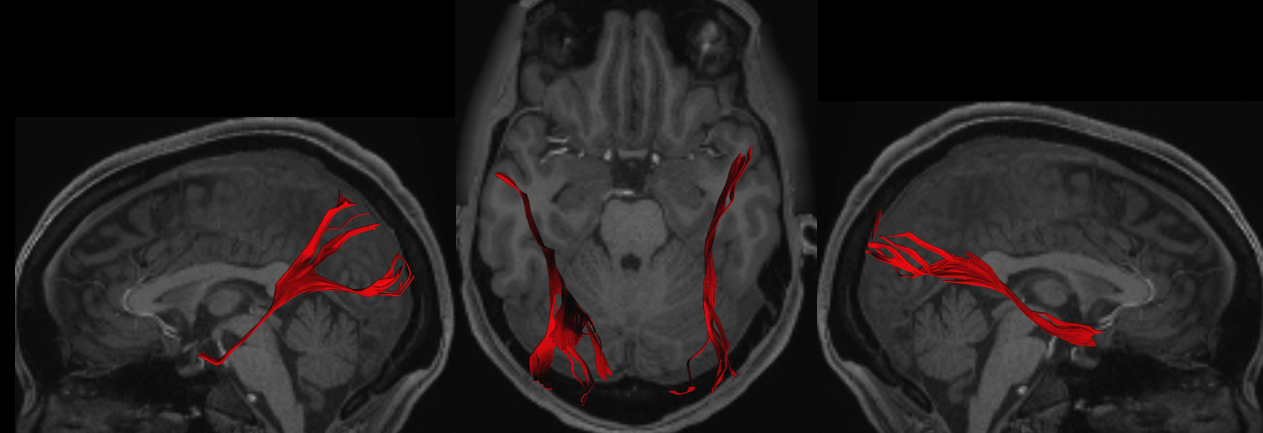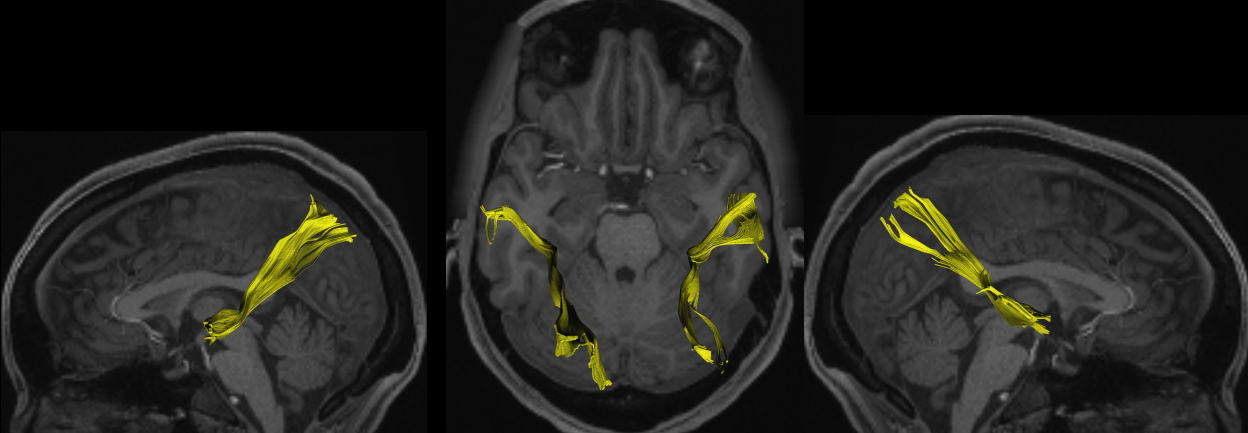

HCP-#24

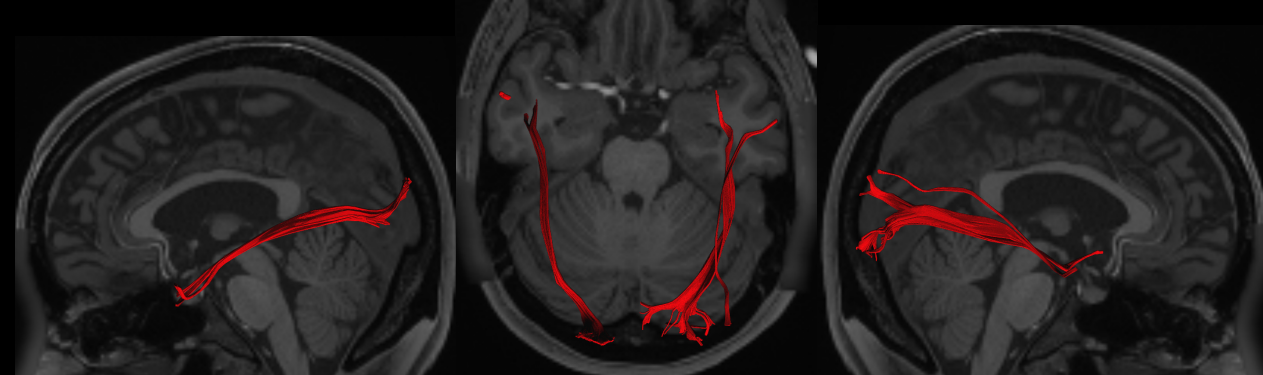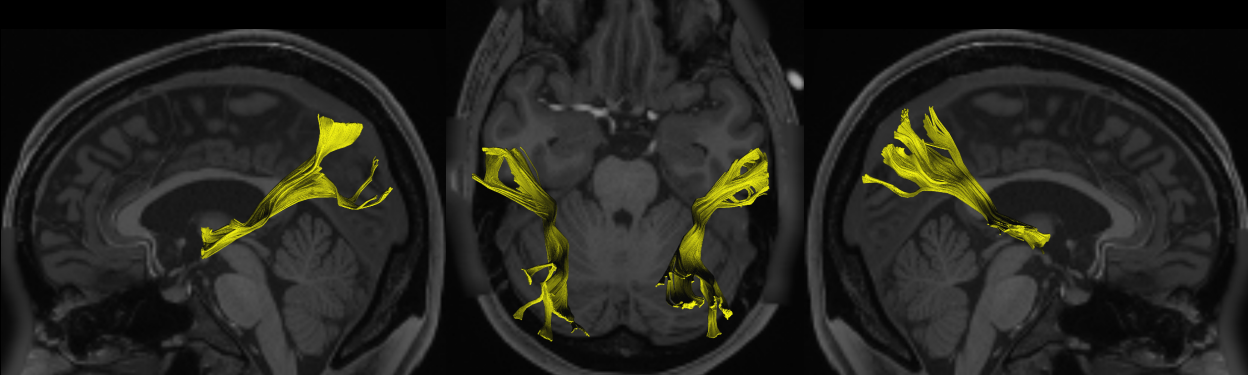

Supplement: Supplementary Material 2 — Tractographic results of the HCP subjects 12–24. The two subsegments of MdLF are displayed in red (anterior segment, aMdLF) and in yellow (posterior segment, pMdLF) on sagittal and axial T1-MR images. L: left side; R: right side. [file Data_Sheet_2.PDF]
